# Lithium carbonate-promoted mixed rare earth oxides as a generalized strategy for oxidative coupling of methane with exceptional yields

**DOI:** 10.1038/s41467-023-43682-5

**Published:** 2023-11-27

**Authors:** Kun Zhao, Yunfei Gao, Xijun Wang, Bar Mosevitzky Lis, Junchen Liu, Baitang Jin, Jacob Smith, Chuande Huang, Wenpei Gao, Xiaodong Wang, Xin Wang, Anqing Zheng, Zhen Huang, Jianli Hu, Reinhard Schömacker, Israel E. Wachs, Fanxing Li

**Affiliations:** 1 North Carolina State University, Campus Box 7905, Raleigh, NC USA; 2 CAS Key Laboratory of Renewable Energy, Guangdong Provincial Key Laboratory of New and Renewable Energy Research and Development, Guangzhou Institute of Energy Conversion, Chinese Academy of Sciences, Guangzhou, China; 3 Institute of Clean Coal Technology, East China University of Science and Technology, Shanghai, China; 4 Department of Chemical and Biological Engineering, Northwestern University, Evanston, IL USA; 5 Operando Molecular Spectroscopy & Catalysis Laboratory, Department of Chemical & Biomolecular Engineering, Lehigh University, Bethlehem, PA USA; 6 Dalian Institute of Chemical Physics, Chinese Academy of Sciences, Dalian, China; 7 Department of Chemical & Biomedical Engineering, West Virginia University, Morgantown, WV USA; 8 Department of Chemistry, Technische Universität Berlin, Straße des 17. Juni 124, Berlin, Germany

**Keywords:** Chemical engineering, Heterogeneous catalysis, Natural gas

## Abstract

The oxidative coupling of methane to higher hydrocarbons offers a promising autothermal approach for direct methane conversion, but its progress has been hindered by yield limitations, high temperature requirements, and performance penalties at practical methane partial pressures (~1 atm). In this study, we report a class of Li_2_CO_3_-coated mixed rare earth oxides as highly effective redox catalysts for oxidative coupling of methane under a chemical looping scheme. This catalyst achieves a single-pass C_2+_ yield up to 30.6%, demonstrating stable performance at 700 °C and methane partial pressures up to 1.4 atm. In-situ characterizations and quantum chemistry calculations provide insights into the distinct roles of the mixed oxide core and Li_2_CO_3_ shell, as well as the interplay between the Pr oxidation state and active peroxide formation upon Li_2_CO_3_ coating. Furthermore, we establish a generalized correlation between Pr^4+^ content in the mixed lanthanide oxide and hydrocarbons yield, offering a valuable optimization strategy for this class of oxidative coupling of methane redox catalysts.

## Abstract (web summary)

Lithium carbonate-promoted mixed rare earth oxides can be used as redox catalysts for OCM at 700 °C and achieve a single-pass C2+ yield up to 30.6%. The high activity is assigned to the peroxide and OH radicals induced by Pr^4+^ in the redox catalyst.

## Introduction

Efficient, single-step conversion of methane into value-added chemicals has been a critical challenge in C1 chemistry. Among the various conversion methods, oxidative coupling of methane (OCM), which employs gas-phase molecular O_2_ to generate higher hydrocarbons (C_2+_) in an autothermal process, has garnered significant research attention since its inception in the 1980s^1^. Over the past 40 years, ~2000 OCM catalysts have been identified for OCM through experimental screening and/or with the assistance of machine learning^1–4^. Among the investigated materials, top-performing candidates predominantly fall within two distinct catalyst families: the unsupported Li-MgO mixed oxide and the supported Mn-Na_2_WO_4_/SiO_2_.

The Li-MgO bulk mixed oxide catalyst was first reported by Lunsford et al. in 1985 and achieved up to 19% C_2+_ yield at 720 °C^5,6^. While various optimizations have been conducted on this catalyst, the C_2+_ yield has yet to exceed 20%^7,8^. Moreover, catalyst deactivation issues persist due to evaporation of lithium in the form of LiOH^9,10^. In 1992, Fang et al. reported that the supported Mn-Na_2_WO_4_/SiO_2_ catalyst demonstrated 23.9% C_2+_ yield at 800 °C^11^. Extensive studies were performed on this catalyst including material screening^12^, surface and bulk structural characterization^13^, reaction pathway and mechanism modeling^14^, and reactor optimization^15^. While deeper mechanistic insights into these catalyst families have been obtained in recent years^14,16,17^, the observed C_2+_ yield has not exceeded 30% for the Mn-Na_2_WO_4_/SiO_2_ catalyst family, which showed satisfactory stability in general. Outside of the Li-MgO and Mn-Na_2_WO_4_/SiO_2_ catalyst families, La_2_O_3_-CeO_2_ nanofibers have exhibited a C_2+_ yield of ~20% at the relatively low temperature of 520 °C^18^. By maximizing all the desired reaction rates and optimizing thermochemistry for all the surface species on an idealized catalyst, Green et al. predicted, through kinetic modeling, that the C_2+_ yield would limit to ~28% in catalytic OCM with O_2_-cofeed^19^. This is consistent with the experimentally reported yields to date.

To address the yield limitations from co-feeding methane and gaseous O_2_, research has also been conducted on spatially and/or temporally separating the contact of methane and O_2_ for OCM. Up to 34.7% C_2+_ yield was reported at 900 °C in diluted methane (*P*_CH4_ < 0.5 atm) using a catalytic membrane reactor composed of a mixed-conductive Ba_0.5_Ce_0.4_Gd_0.1_Co_0.8_Fe_0.2_O_3−δ_ membrane and a supported Mn-Na_2_WO_4_/SiO_2_ catalyst. However, rapid membrane degradation was observed, a common challenge for membrane-based OCM at such elevated temperatures^20^. Lattice oxygen-based OCM has also been performed under a chemical-looping (CL) mode, which utilizes a reducible metal oxide operated through cyclic redox steps under alternating methane and O_2_ environments^21,22^. Gaffney et al. pioneered the concept of chemical looping-OCM (CL-OCM) and reported that a Na impregnated Pr_6_O_11_ catalyst achieved up to 16% C_2+_ yield at 775 °C^23^. The chemical looping mode utilizes the redox between Pr^4+^ and Pr^3+^ and demonstrated ~4% higher C_2+_ yield than the O_2_-cofeed mode. More recently, Fan et al. proposed the idea of CL-OCM by designing a Li and W co-doped Mg_6_MnO_8_ redox catalyst that exhibited 28.6% C_2+_ yield at 850 °C^24,25^. To date, more than 10,000 articles have been published on OCM. However, none of the prior studies have demonstrated >30% C_2+_ yield with satisfactory stability. Moreover, most of these studies were carried out with highly diluted methane, which would not be suitable for practical applications. Based on experimental data coupled with kinetic analyses, Labinger et al. argued that higher methane partial pressures would lead to severe yield penalties^26^. On the other hand, it has been estimated that for OCM to achieve commercial viability, a C_2+_ yield exceeding 30–35% at practical partial pressures (~1 atm) is required^27^. As such, a gap clearly exists between reported academic research results and industrial application^28^.

From a mechanistic aspect, various active sites or active species have been postulated to be responsible for methane activation. Taking Li-MgO as a model catalyst, early studies by Lunsford et al. suggested that Li^+^O^−^ is the active site based on electron spin resonance (EPR) of quenched catalysts in the presence of O_2_ with the g_⊥_ = 2.054 signal^5,29^. Based on the O 1*s* shoulder observed at 533 eV in ex-situ X-ray photoelectron spectroscopy (XPS) using a spectrometer equipped with a pretreatment chamber, Stair et al. argued for the presence of peroxide or Li^+^O^−^ species in the surface region (<3 nm)^30^. The presence of peroxides for OCM on a related, unsupported Ba/MgO mixed oxide catalyst was reported by Lunsford et al. on the basis of in-situ Raman (with the BaO_2_ band at 842 cm^−1^) and ex-situ XPS (with the O 1 *s* peak at 531 eV)^31,32^. While the presence of peroxides was largely confirmed, the necessity for Li^+^O^−^ sites was questioned by subsequent studies involving both experimental work and quantum chemistry calculations^33,34^. These studies argued that Mg^2+^O^2−^ sites or defective MgO surfaces are responsible for methane activation, and Li only acts as a structural modifier instead of an active center^33,34^. The search for active sites in the supported Mn-Na_2_WO_4_/SiO_2_ was similarly challenging. A number of earlier studies, mostly through ex-situ measurements, proposed that the active sites are either Na-O-Mn, Na-O-W or other bonds belonging to bulk crystalline phases selected from the Mn-Na-W-O components^35^. More recently, Wachs et al. conducted in-situ Raman studies and demonstrated that none of the abovementioned crystalline phases are actually present at the OCM reaction temperature (900 °C), and the active site for methane activation are isolated, pseudotetrahedral Na-coordinated WO_4_ surface sites (Na-WO_4_) on the SiO_2_ support^13,14,16^. Through separate studies, Takanabe and Tao et al. detected the presence of peroxide species for both supported K_2_WO_4_/SiO_2_ and Na_2_WO_4_/SiO_2_^36,37^ catalysts with in-situ XPS. Using laser induced fluorescence (LIF) measurements. Tao further proposed that the presence of near-surface peroxides can lead to the formation of hydroxyl radicals for methane activation.

Given the potential role of surface/subsurface peroxide species and the redox properties of praseodymium oxides in the context of chemical looping^23^, the current study focuses on Pr-containing lanthanide oxides with a Li_2_CO_3_ promoter for CL-OCM. Li_2_CO_3_ was selected because it has good ${{{{{{\rm{O}}}}}}}_{2}^{2-}$ solubility and conductivity, and was previously reported to be effective for ethane activation^38^. Unsupported bulk mixed oxides containing Pr and another lanthanide cation, on the other hand, can beneficially modify the redox properties of Pr^4+^/Pr^3+^ ^23^. In the present study, a series of Pr-containing lanthanide oxides with a thin surface film of Li_2_CO_3_ (LnPrO_3+x_@Li_2_CO_3_, Ln = La, Eu, Ho, Dy, Sm, and Nd) for CL-OCM were synthesized and characterized. This family of materials exhibited up to 30.6% single-pass C_2+_ yield with stable performance at 700 °C. The roles of the mixed oxide core and Li_2_CO_3_ shell, as well as the interplays among the Pr oxidation state, active peroxide formation upon Li_2_CO_3_ coating, and OCM performance were determined by ex-situ X-ray absorption near edge structure (XANES), in-situ Raman, in-situ X-ray diffraction (XRD), in-situ XPS, and quantum chemistry calculations.

## Results

### Structures of catalyst bulk phase and surface region under different environments

**Fig. 1:**
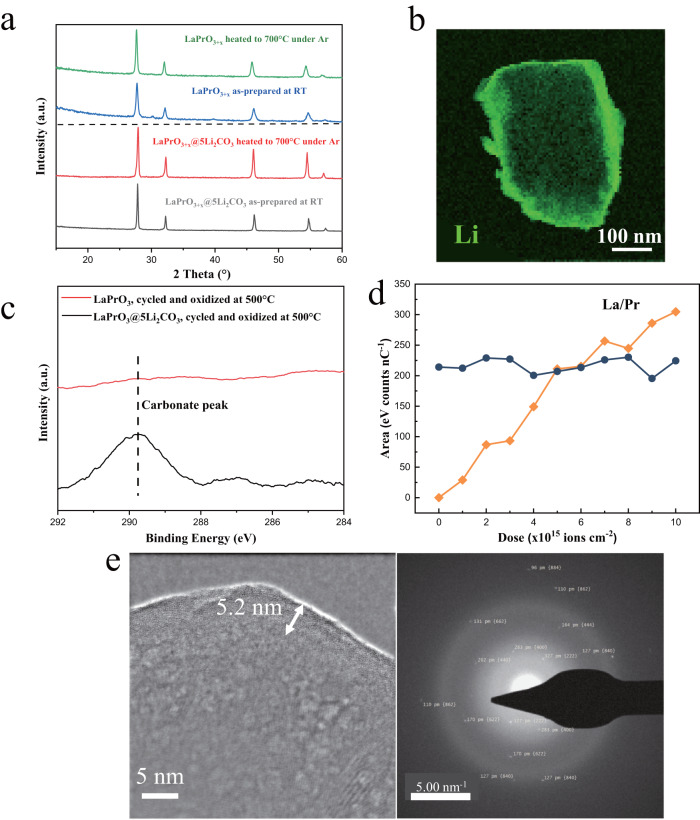
Ex-situ and in-situ spectroscopic characterizations for LaPrO_3+x_@5Li_2_CO_3_. **a** In-situ XRD on LaPrO_3+x_@5Li_2_CO_3_ under air at 700 °C; **b** Ex-situ TEM-EELS on LaPrO_3+x_@5Li_2_CO_3_ in vacuum; **c** In-situ XPS on LaPrO_3+x_ and LaPrO_3+x_@5Li_2_CO_3_, both LaPrO_3+x_ and LaPrO_3+x_@5Li_2_CO_3_ were reduced with diluted methane at 700 °C and re-oxidized with diluted oxygen at 500 °C in the in-situ XPS chamber; **d** Quasi in-situ HS-LEIS on LaPrO_3+x_@5Li_2_CO_3_ treated in 600 °C under 10% O_2_ in a pretreatment chamber; **e** In-situ TEM and electron diffraction of LaPrO_3+x_@5Li_2_CO_3_ at 700 °C under diluted O_2_.

While all the Li_2_CO_3_ promoted LnPrO_3+x_ oxides (Ln = La, Eu, Ho, Dy, Sm, Nd) were active for OCM (as will be discussed in later sections), LaPrO_3+x_@5Li_2_CO_3_ (5 refers to 5 wt.% Li_2_CO_3_ loading) was selected as a representative redox catalyst for detailed characterizations since it showed excellent performance and La is a relatively abundant rare earth element. The LaPrO_3+x_@5Li_2_CO_3_ redox catalyst consists of a core-shell structure. The core consists of the crystalline LaPrO_3+x_ bulk phase as shown by in-situ XRD at 700 °C (Fig. 1a). The crystalline LaPrO_3+x_ core is covered by a thin Li_2_CO_3_ shell as revealed by (i) spatial distribution of Li in the mapping of the ex-situ TEM-EELS analysis on a catalyst particle (Fig. 1b), (ii) surface enrichment of carbon with TEM-EDS (Supplementary Fig. S1) whereas carbonate-free LaPrO_3+x_ does not exhibit a XPS signal for carbon (Fig. 1c), (iii) presence of carbon and lithium in the surface region of LaPrO_3+x_@5Li_2_CO_3_ with in-situ XPS (Supplementary Fig. S2), and (iv) absence of Pr and La on the outermost surface layer (0.3 nm) as revealed by high sensitivity - low energy ion scattering (HS-LEIS) analysis of the surface, and the increase in the La and Pr signals with sputtering depth (Fig. 1d). The thin Li_2_CO_3_ shell (<5 nm) is amorphous and lacks long range order. Therefore, its signal does not appear in the in-situ XRD pattern (Fig. 1a). Furthermore, bulk Li_2_CO_3_ melts at 723 °C, suggesting that the surface layer is likely be in a molten state under the OCM reaction conditions (~700 °C) given the lower melting temperatures of thin films^38^. The in-situ TEM analysis (Fig. 1e) further verifies the morphology and composition of the LaPrO_3+x_@5Li_2_CO_3_ catalyst, which consists of a crystalline LaPrO_3+x_ core enveloped by a thin amorphous Li_2_CO_3_ shell at 700 °C.

### The relationship between near-surface peroxide and Pr^4+^

**Fig. 2:**
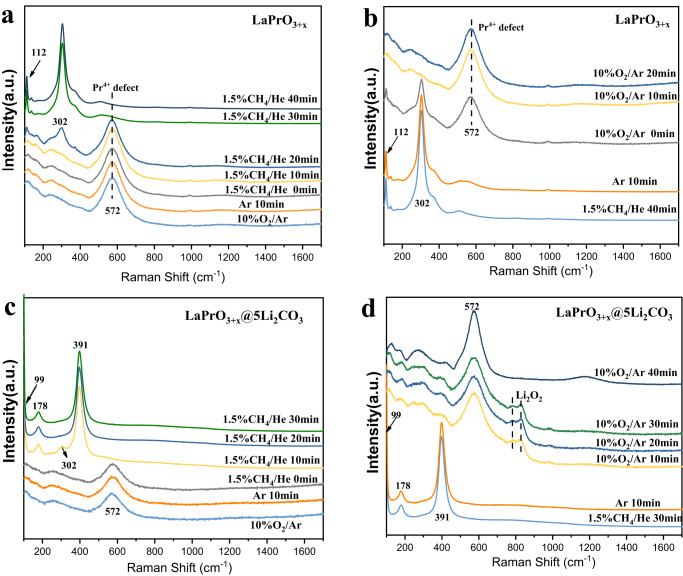
In-situ Raman for LaPrO_3+x_ and LaPrO_3+x_@5Li_2_CO_3_. In-situ Raman for **a** LaPrO_3+x_ reduction at 700 °C, **b** LaPrO_3+x_ reoxidation at 700 °C, **c** LaPrO_3+x_@5Li_2_CO_3_ reduction at 700 °C, **d** LaPrO_3+x_@5Li_2_CO_3_ reoxidation at 700 °C.

Given that CL-OCM reactions proceed through cyclic removal (OCM step) and replenishment (re-oxidation step) of lattice oxygen from bulk reducible oxides in the redox catalyst, the dynamics of the bulk LaPrO_3+x_ phase for LaPrO_3+x_ and LaPrO_3+x_@5Li_2_CO_3_ under oxidizing and methane reducing conditions were further monitored with in-situ XRD, Raman and XPS at 700 °C. The oxidized Li-free bulk LaPrO_3+x_ mixed oxide is present as cubic-LaPrO_3.33_ and transforms to a mixture of cubic-La_2_O_3_ and cubic-Pr_2_O_3_ after methane reduction (see in-situ XRD in Supplementary Fig. S3). This is corroborated by the corresponding in-situ Raman spectra (Fig. 2a, b), showing that the oxidized cubic-LaPrO_3.33_ phase (572 cm^−1^) is reduced to cubic-La_2_O_3_ and cubic Pr_2_O_3_ (112 and 302 cm^−1^). Westermann et al. assigned the band at 572 cm^−1^ to Pr^4+^ defects^39^. Re-oxidation converts the reduced phase back to the initial oxidized state. Cubic-LaPrO_3+x_ is also present in the oxidized LaPrO_3+x_@5Li_2_CO_3_ catalyst, but it reversibly transforms to the bulk hexagonal-LaPrO_3+x_ phase after methane reduction. This is confirmed by in-situ XRD in Supplementary Fig. S3 and in-situ Raman spectra in Fig. 2c, d, where the oxidized bulk c-LaPrO_3+x_ (572 cm^−1^) was reduced to the bulk h-LaPrO_3+x_ (178 and 392 cm^−1^). Thus, the amorphous Li_2_CO_3_ shell affects the structure of the bulk LaPrO_3+x_ phase under the OCM conditions. The presence of the Li_2_CO_3_ shell also resulted in the formation of peroxide species (O_2_^2−^: in-situ Raman band at ~850 cm^−1^ characteristic of Li_2_O_2_)^40^ during the transient oxidation of the reduced LaPrO_3+x_@5Li_2_CO_3_ mixed oxide. The absence of peroxide species for the Li-free LaPrO_3+x_ and the presence of the peroxide species for LaPrO_3+x_@5Li_2_CO_3_ suggest that the peroxide species are associated with the thin Li_2_CO_3_ shell. It is noted that the surface area of Li_2_CO_3_ promoted LaPrO_3+x_ is quite low (~1 m^2^/g, Supplementary Table S1 summarizes the surface areas of bare LaPrO_3+x_ and LaPrO_3+x_ with different Li_2_CO_3_ loadings), the ability for Raman to detect the peroxide species in this low surface area assay suggests that the detected peroxide signal cannot just be surface bound. Rather, contributions from bulk peroxide species, e.g. peroxides dissolved/incorporated in the amorphous Li_2_CO_3_ shell, is more likely. We also note that Raman did not detect Li_2_CO_3_ peaks from this sample, this is probably due to its low loading (5 wt.%) and the peak broadening effect of amorphous carbonate as the temperature increases^41^. This peak broadening effect was confirmed via an in-situ Raman experiment on LaPrO_3+x_@5Li_2_CO_3_ under 5%CO_2_ (balance Ar) with temperature ramping up from 120 to 700 °C. As shown in Supplementary Fig. S4a, LaPrO_3+x_@5Li_2_CO_3_ exhibited a clear surface carbonate peak between 1100–1300 cm^−1^. This peak, however, tends to be broadened and smoothed out when the temperature gradually ramped up to 700 °C. We note that this broadening effect is not likely due to the thermal decomposition of Li_2_CO_3_ since the presence of 5 vol.% CO_2_ would inhibit carbonate decomposition from a thermodynamic standpoint. We have also compared ex-situ Raman under air in room temperature for LaPrO_3+x_@3Li_2_CO_3_, LaPrO_3+x_@5Li_2_CO_3_ and LaPrO_3+x_@10Li_2_CO_3_. All these samples exhibited surface carbonate peaks of similar relative intensities (Supplementary Fig. S4b). Thus, the absence of surface carbonate peaks for LaPrO_3+x_@5Li_2_CO_3_ under in-situ Raman is more likely due to temperature effect rather than the Li_2_CO_3_ loading effect.

**Fig. 3:**
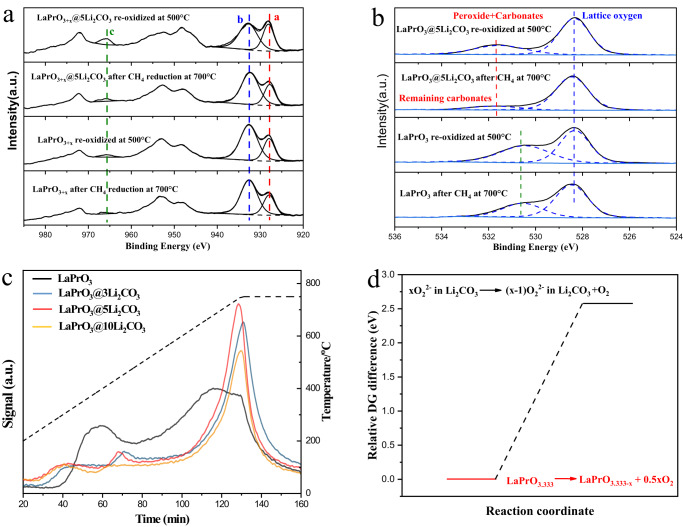
Probe of oxygen species evolution. In-situ XPS spectra on LaPrO_3+x_ and LaPrO_3+x_@5Li_2_CO_3_ after methane reduction and oxidation treatments: methane reduction was conducted at 700 °C and re-oxidation was conducted at 500 °C: **a** and **b** show Pr 4*f* and O 1*s* peaks, respectively; **c** O_2_-TPD from LaPrO_3+x_ and LaPrO_3+x_@Li_2_CO_3_ with different Li_2_CO_3_ loadings; **d** Relative ∆G difference of O_2_ release from LaPrO_3.33_ and Li_2_O_2_ in amorphous Li_2_CO_3_.

Our previous study on chemical looping ethane conversion indicated that the formation of peroxide species from mixed oxides can be linked to the presence of highly reducible cation components^38^. Therefore, the oxidation states of the Pr cations in the oxidized LaPrO_3+x_ and LaPrO_3+x_@5Li_2_CO_3_ mixed oxide catalysts were determined by ex-situ XANES first. It was shown that both Pr^3+^ and Pr^4+^ are present for the LaPrO_3+x_ and LaPrO_3+x_@5Li_2_CO_3_ catalysts (Supplementary Fig. S5)^42^. It is evident that LaPrO_3+x_@5Li_2_CO_3_ contains more bulk Pr^4+^ than LaPrO_3+x_. The corresponding in-situ XPS measurement also detected the presence of near-surface Pr^4+^ and peroxide species. Figure 3a shows the Pr 3d XPS spectra of LaPrO_3+x_ and LaPrO_3+x_@5Li_2_CO_3_. Although the quantification of Pr^4+^/Pr^3+^ with XPS is complex, Sinev et al. have reported the characteristic peaks and features for Pr^4+^ with in-situ XPS by switching between oxidizing and reducing atmospheres on a Pr-Ce mixed oxide^43^. A large “a/b” peak area ratio and a large “c” peak area are representative of the Pr^4+^ features (as labeled in Fig. 3a). As shown in Fig. 3a, LaPrO_3+x_@5Li_2_CO_3_ exhibited much more intense Pr^4+^ features than LaPrO_3+x_ in its oxidized state, confirming the abundance of Pr^4+^ in the near surface region in the presence of the Li_2_CO_3_ coating. It is noteworthy that Pr^4+^ in the near surface region of LaPrO_3+x_@5Li_2_CO_3_ is largely transformed into Pr^3+^ after contacting methane, as indicated by the decreased “c” peak area and “a/b” peak area ratio. In comparison, the changes in the Pr^4+^ features were quite unremarkable in Li-free LaPrO_3+x_ when exposed to methane. This is likely related to spontaneous decomposition of LaPrO_3+x_ via the reduction of Pr^4+^ in the surface region without the Li_2_CO_3_ layer, under the low oxygen partial pressure in the in-situ XPS (~1 mbar). The corresponding in-situ XPS O 1*s* spectra are presented in Fig. 3b. LaPrO_3+x_ exhibited an O 1*s* peak at B.E. = at 528.2 eV that is assigned to lattice oxygen species. The shoulder O 1*s* peak at B.E. = 530.7 eV for LaPrO_3+x_ has not been reported previously. It is thought to arise from stable hydroxyls to the bare LaPrO_3+x_ since it is independent of the reduction and oxidation treatments. LaPrO_3+x_@5Li_2_CO_3_ showed two XPS O 1*s* peaks: B.E. = 528.2 eV, which corresponds to lattice oxygen and does not vary substantially upon reduction or oxidation; and B.E. = 531.8 eV, which is consistent with previous literature assignments of peroxide^37^. This assignment for peroxide is further substantiated by the fact that it decreased substantially when contacting methane, and increased upon re-oxidation with O_2_. In-situ FTIR-DRIFTS on LaPrO_3+x_@5Li_2_CO_3_ further confirmed that the carbonate peak increased when methane was injected onto the sample (Supplementary Fig. S6). The abundance of peroxide species in LaPrO_3+x_@5Li_2_CO_3_, and their absence in LaPrO_3+x_, can be explained by: (a) the presence of the Li_2_CO_3_/Li_2_O layer, which inhibits peroxide decomposition into molecular O_2_ from the surface of LaPrO_3+x_ and (b) the increased presence of Pr^4+^ compared to LaPrO_3+x_ as confirmed by XANES and in-situ XPS, which favors O_2_^2-^ formation.

The stoichiometric x value in Li-free LaPrO_3+x_ was determined to be 0.33 by thermogravimetric analysis (TGA) upon methane reduction at 700 °C (Supplementary Fig. S7). O_2_-TPD were further conducted on LaPrO_3+x_ and LaPrO_3+x_@5Li_2_CO_3_ (Fig. 3c), showing that LaPrO_3+x_ exhibited substantial O_2_ release at much lower temperatures than LaPrO_3+x_@5Li_2_CO_3_. Meanwhile, LaPrO_3+x_@Li_2_CO_3_ with 3–10 wt.% Li_2_CO_3_ loadings all exhibited a primary O_2_ release peak at ~750 °C. The suppressed peroxide decomposition to gaseous molecular O_2_ was further examined via ab-initio molecular dynamics (AIMD). As shown in Fig. 3d, O_2_ formation from LaPrO_3+x_ without the Li_2_CO_3_ shell is far more facile than gaseous molecular O_2_ formation from Li_2_O_2_ in the amorphous Li_2_CO_3_ thin film. The detailed structural changes of both reactions are shown in Supplementary Fig. S8. This is consistent with the higher oxygen release peak temperature in Fig. 3c and indicates that the Li_2_CO_3_ layer stabilizes the O_2_^2−^ peroxide species formed from LaPrO_3+x_. Furthermore, Li_2_CO_3_ has been shown to have a substantial solubility of O_2_^2−^ peroxide species^38^.

### Active redox species and reaction pathway

**Fig. 4:**
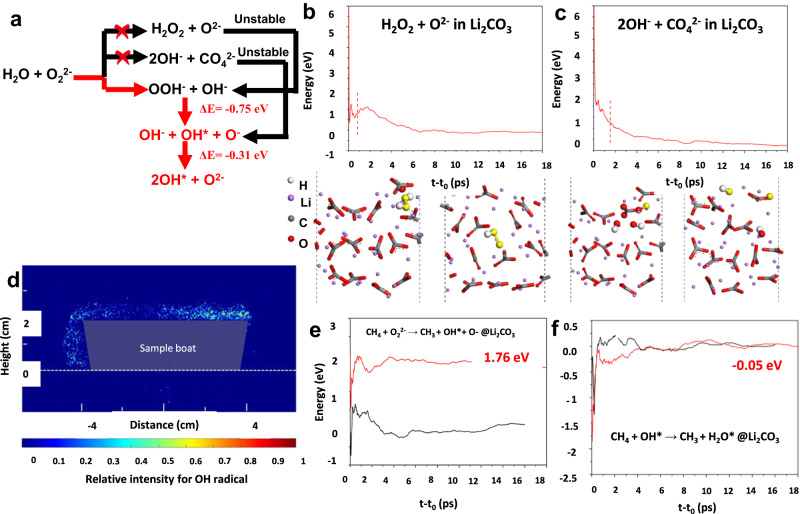
Probe of OH radical evolution. **a** Summary of the possible reaction product of H_2_O + O_2_^2−^; **b**, **c**: Mean energies as a function of elapsed time (*t-t*_*0*_) for evolution of H_2_O_2_ + O^2−^ and OH^−^ + CO_4_^2−^ in molten Li_2_CO_3_, respectively. The electrophilic oxygen atoms that are involved in the reactions are highlighted in yellow to provide better visualization; **d** LIF experiments on SiO_2_@5Li_2_CO_3_, scale bar shows the relative intensity for OH radical; **e** and **f**:, respectively.

A detailed AIMD study was conducted to determine the fate of peroxide in Li_2_CO_3_. Interaction between O_2_^2−^ and water has been reported to yield OH radicals (OH^*^) in OCM reactions^37^. We further investigated groups of possible products and corresponding reaction pathways using AIMD. As can be seen in Fig. 4a, peroxide is more favorable to evolve into hydroxyl radicals by interacting with H_2_O dissolved in the salt. Clearly, H_2_O_2_ + O^2−^ and 2OH^−^ + CO_4_^2−^ cannot be stable because they spontaneously convert to OOH^−^ + OH^−^ (Fig. 4b) and OH^−^ + OH^*^ + O^−^ (Fig. 4c), respectively. It was calculated that OH^−^ + OH^*^ + O^−^ can further evolve into 2OH^*^ + O^2−^, intensifying OH^*^ production. This was confirmed with LIF experiments on Li_2_CO_3_ coated SiO_2_ as a model material at 700°C under O_2_ and steam, which can detect the formation of OH^*^ (Fig. 4d). The interactions between active species and methane were further studied. It was demonstrated that direct C-H bond activation in methane by O_2_^2−^ is not energetically favorable (Fig. 4e). In comparison, the as-formed hydroxyl radical is highly active for methane activation (Fig. 4f). This is also consistent with previous literature report on Mn-Na_2_WO_4_/SiO_2_ catalysts^44^. Based on the abovementioned experimental and simulation results, the reaction pathway involves peroxide formation on the LaPrO_3+x_ surface resulting from Pr^4+^ → Pr^3+^ transition, dissolution of the O_2_^2−^ in the carbonate phase, and subsequent hydroxyl radical formation and CH_3_ radical formation by C-H bond cleavage. The surface initiated radical reaction will further drive C_2+_ formation in the gas phase^45^.

### Catalyst reactivity performance for OCM

**Fig. 5:**
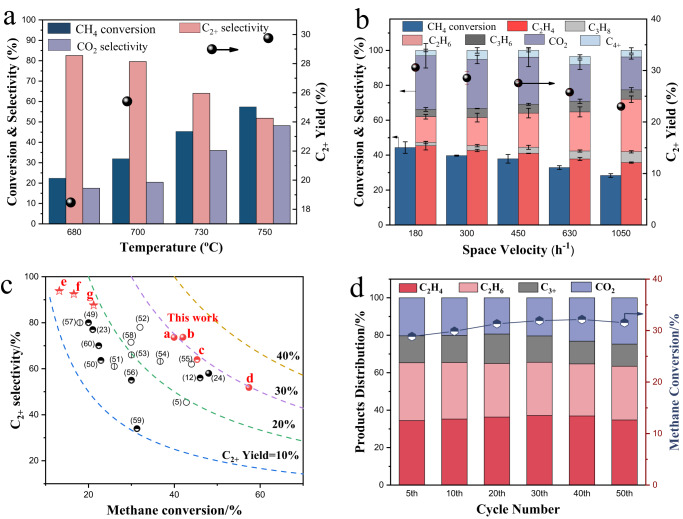
Li_2_CO_3_ coated LaPrO_3+x_ as a redox catalyst for CL-OCM. Catalyst performance for CL-OCM: **a** Temperature effect for LaPrO_3+x_@5Li_2_CO_3_: *P*_CH4_ = 0.4, *GHSV* = 1050 h^−1^; **b** Space velocity effect for LaPrO_3+x_ @5Li_2_CO_3_: *T* = 700 °C, *P*_CH4_ = 1.0; The error bars were expressed as the standard deviations of triplicate experiments. **c** Comparison of LaPrO_3+x_@5Li_2_CO_3_ with previously reported OCM catalysts: full-filled, half-filled and empty dots represent *P*_CH4_ = 0.8–1, 0.4–0.8 and <0.4 atm, respectively. Seven data points from this work are included: **a** *T* = 700 °C, *P*_CH4_ = 1.0 atm, *GHSV* = 180 h^−1^; **b** *T* = 700 °C, *P*_CH4_ = 1.0 atm, *GHSV* = 300 h^−1^; **c** *T* = 730°C, *P*_CH4_ = 0.4 atm, *GHSV* = 1050 h^−1^; **d** *T* = 750°C, *P*_CH4_ = 1.0 atm, *GHSV* = 180 h^−1^; **e**–**g** stands for a Mn-Na_2_WO_4_/SiO_2_ catalyst tested under a redox mode at *P*_CH4_ = 0.4 atm and *GHSV* = 1050 h^−1^, with reaction temperature = 700, 750 and 800° respectively; **d** 50-redox cycle test on LaPrO_3+x_@5Li_2_CO_3_: *T* = 700 °C, *P*_CH4_ = 0.4, *GHSV* = 1050 h^−1^.

The Li-free LaPrO_3+x_ exhibited 57.6% methane conversion, but only 5.14% C_2+_ selectivity with CO_2_ as the main product at 700°C and 1050 hr^−1^ gas hourly space velocity (GHSV). Li_2_CO_3_ promotion significantly increases the C_2+_ selectivity of LaPrO_3+x_. Figure 5 summarizes the effects of reaction temperature, space velocity, and methane partial pressure for LaPrO_3+x_@5Li_2_CO_3_. Higher temperature led to higher methane conversion and C_2+_ yield, but with increased CO_2_ selectivity (Fig. 5a). At 700 °C under pure methane (P_CH4_ = 1 atm), decreasing GHSV led to increased methane conversions, with only a slight decrease in C_2+_ selectivity (Fig. 5b). A maximum C_2+_ yield of 30.6% was obtained at 180 h^−1^, the lowest GHSV tested due to instrumentation limitations. The effect of the OCM step duration was investigated, with the optimum duration determined to be 60 s under the current reactor setting. Longer OCM steps decreased methane conversion, while shorter steps reduced C_2+_ selectivity (Supplementary Fig. S9). We note that the results at all GHSV investigated exceeded the generally accepted “100% rule” for OCM, namely that the sum of methane conversion and C_2+_ selectivity does not exceed 100%^46^. The ability to use undiluted methane represents another advantage from a practical standpoint, when compared to most of the previous literature studies that employed significant amounts of diluent. The effect of methane partial pressure was further illustrated in Supplementary Fig. S10. As can be seen, the space-time yield for ethane, ethylene and CO_2_ increased almost linearly with increased methane partial pressure from 0.2 atm to 1.5 atm. This suggests a first-order kinetics for both C_2_ and CO_x_ formation. Therefore, the LaPrO_3+x_@5Li_2_CO_3_ redox catalyst can operate at elevated methane partial pressures, which would be highly beneficial for downstream separation and processing of the C_2+_ products. We also note that many of the previously reported OCM catalysts suffered from severe yield penalty at elevated methane partial pressures^26,47,48^. These findings highlight the advantages of LaPrO_3+x_@5Li_2_CO_3_ in CL-OCM. Figure 5c compares LaPrO_3+x_@5Li_2_CO_3_ with previously reported OCM catalysts^49–60^: LaPrO_3+x_@5Li_2_CO_3_ exhibited the highest OCM yield reported so far, and is the only catalyst that exceeds the 30% single-pass C_2+_ yield at 100% methane partial pressure. The optimal operating temperature at 700 °C is also significantly lower than the classical OCM catalysts such as Mn-Na_2_WO_4_/SiO_2_, which exhibits optimal performance at ~850 °C. Traditional Mn-Na_2_WO_4_/SiO_2_ catalyst was also synthesized and tested under redox OCM to compare with LaPrO_3+x_@5Li_2_CO_3_. C_2+_ yields of 12.4%, 15.4% and 18.6% were observed at 700, 750 and 800 °C respectively, indicating that LaPrO_3+x_@5Li_2_CO_3_ is superior especially at lower reaction temperatures. The LaPrO_3+x_@5Li_2_CO_3_ catalyst exhibited excellent catalyst stability, as confirmed by long-term performance tests at 700 °C and 1050 h^−1^ GHSV. Both methane conversion and C_2+_ selectivity were stable within 50 redox cycles as shown in Fig. 5d. In comparison, Li/MgO tends to deactivate after contacting methane and O_2_ at 750 °C^34^. The high stability of LaPrO_3+x_@5Li_2_CO_3_ is ascribed to the preservation of the amorphous Li_2_CO_3_ overlayer. This was confirmed via ex-situ XPS. As can be seen in Supplementary Fig. S11, the carbonate O 1*s* peak portion for LaPrO_3+x_@5Li_2_CO_3_ does not decrease after redox cycles, indicating that surface Li_2_CO_3_ is maintained. This was also separately validated via a TGA based cyclic experiment (Supplementary Fig. S12). This is substantially different from literature reports on Li/MgO, where Li content decreased from 3.1 wt.% to ~0.1 wt.% within 20 h^61^. The preservation of Li in LaPrO_3+x_@5Li_2_CO_3_ is likely due to the lower reaction temperature and the abundance of Li_2_CO_3_ relative to LiOH, thereby inhibiting Li evaporation^10^. Carbon deposition was negligible after the long-term cycle, as proven by the absence of CO and CO_2_ during the re-oxidation step (Supplementary Fig. S13). We also note that under the reaction temperature, Li_2_CO_3_ could partially decompose into Li_2_O, while the as-formed Li_2_O can react with the by-product CO_2_ in the OCM step and re-form Li_2_CO_3_. Thus, the catalyst surface is likely to be in a mixed state of Li_2_CO_3_ and Li_2_O at any reaction stage. This is proven by using LiNO_3_ instead of Li_2_CO_3_ for wet impregnation onto LaPrO_3+x_, while keeping the same Li amount. The as-synthesized LaPrO_3+x_@Li_2_O (after nitrate decomposition) started to exhibit activity for OCM after a few reaction cycles, although the C_2+_ yield is lower than that of LaPrO_3+x_@Li_2_CO_3_ (Supplementary Fig. S14). The presence of Li_2_O in Li_2_CO_3_ can be beneficial for the formation of Li_2_O_2_ by reacting with the active oxygen species on the LaPrO_3+x_ surface.

### Generalizability of the OCM catalyst design strategy

**Fig. 6:**
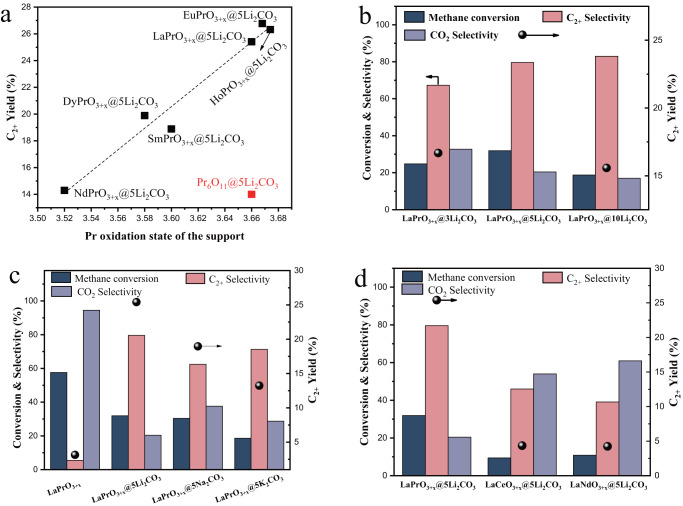
Extension of the LaPrO_3+x_@5Li_2_CO_3_ redox catalyst. **a** Plots of C_2+_ yields with Pr oxidation states of different mixed lanthanide oxide supports; **b** Redox OCM performance comparison of LaPrO_3+x_@3Li_2_CO_3_, LaPrO_3+x_@5Li_2_CO_3_ and LaPrO_3+x_@10Li_2_CO_3_; **c** Redox OCM performance comparison of LaPrO_3+x_, LaPrO_3+x_@Li_2_CO_3_, LaPrO_3+x_@5Na_2_CO_3_ and LaPrO_3+x_@5K_2_CO_3_; **d** Redox OCM performance comparison of LaPrO_3+x_@5Li_2_CO_3_, LaCeO_3+x_@5Li_2_CO_3_ and LaNdO_3+x_@5Li_2_CO_3_. Reaction conditions for **b**–**d**: *T* = 700 °C, *P*_CH4_ = 0.4 atm, *GHSV* = 1050 h^−1^.

The core-shell redox catalyst design strategy can be extended to other Pr-containing mixed lanthanide oxides. NdPrO_3+x_, DyPrO_3+x_, SmPrO_3+x_, HoPrO_3+x_ and EuPrO_3+x_ were synthesized using a similar method. The Pr oxidation states of the carbonate-free mixed oxides were determined by TGA measurement of oxygen stoichiometry upon methane reduction at 700 °C. The mixed oxides were then loaded with 5 wt.% Li_2_CO_3_ and examined for OCM. The C_2+_ yields, plotted in Fig. 6a, correspond to the Pr oxidation state in the mixed oxides. A linear relationship is observed between the C_2+_ yield and the initial Pr oxidation state. EuPrO_3+x_@5Li_2_CO_3_ and HoPrO_3+x_@5Li_2_CO_3_ even achieved slightly better C_2+_ yields than LaPrO_3+x_@5Li_2_CO_3_. The significantly reduced C_2+_ yield of Pr_6_O_11_@5Li_2_CO_3_, despite the Pr oxidation state in Pr_6_O_11_ being +3.67, can be attributed to the instability of the Pr_6_O_11_ phase in the presence of Li_2_CO_3_. This leads to the formation of the Li_26_Pr_36_O_73_ phase after cycling. (see Supplementary Fig. S15a) and consistent with the report by Aono et al., who observed the same phase by heating up the a Pr_6_O_11_ and Li_2_CO_3_ mixture^62^. The decrease in the Pr oxidation state and disruption of the Li_2_CO_3_ layer renders low C_2+_ yield. In comparison, all the Li_2_CO_3_ promoted mixed lanthanide oxides, which the exception of NdPrO_3+x_, maintained their original phases after cycling, and no Li-containing phases were observed (Supplementary Figs. S15b–e). This also highlights the importance of the secondary rare earth metal cation such as La and Sm, which stabilizes Pr^4+^ and inhibits the solid-state reaction with the Li_2_CO_3_ promoter. The Li_2_CO_3_ loading effect on LaPrO_3+x_ was also investigated as shown in Fig. 6b. Although the oxygen release behaviors were all substantially altered with different Li_2_CO_3_ loadings as shown in O_2_-TPD (Fig. 3c), the loading amount exhibits an optimum and LaPrO_3+x_@5Li_2_CO_3_ achieved the highest C_2+_ yield. In addition to Li_2_CO_3_, other alkali metal carbonate promoters including Na_2_CO_3_ and K_2_CO_3_ were also investigated as shown in Fig. 6c. Switching from Li_2_CO_3_ to Na_2_CO_3_ and K_2_CO_3_ leads to decreased catalyst activities and decreased C_2+_ yields. This may be due to the lower activities of Na_2_O_2_ and K_2_O_2_, where Na_2_O_2_ and K_2_O_2_ are more thermodynamically stable than Li_2_O_2_ (Supplementary Table S2). The necessity of Pr in the mixed metal oxide support was also investigated by switching Pr to Ce and Nd, where Ce and Nd locating very close to Pr in the periodic table in the lanthanide family. As shown in Fig. 6d, both LaCeO_3+x_@5Li_2_CO_3_ and LaNdO_3+x_@5Li_2_CO_3_ exhibited very low C_2+_ yields and high selectivities towards CO_2_. This is probably due to the properties of the Pr^4+^↔Pr^3+^ redox pair, which leads to efficient generation of peroxide oxygen species in the Li_2_CO_3_ salt.

## Discussion

In this work, we present a generalized strategy for the chemical looping - oxidative coupling of methane (CL-OCM) using Li_2_CO_3_-promoted mixed rare earth oxides. A detailed study on LaPrO_3+x_@Li_2_CO_3_ revealed a single-pass C_2+_ yield of up to 30.6% with good catalyst stability at 700°C. Additionally, the operational partial pressure of methane can exceed 1 atm, offering potential industrial benefits. The Li_2_CO_3_ promotion formed a surface layer on LaPrO_3+x_, increasing both the bulk and surface Pr^4+^ contents, which in turn enhanced OCM activity. This finding was corroborated by ex-situ XANES, in-situ Raman, in-situ XRD, and in-situ XPS analyses. In-situ Raman and XPS measurements also suggested that Pr^4+^ contributed to the presence of near surface peroxide on LaPrO_3+x_@Li_2_CO_3_. The peroxide species would subsequently transform into hydroxyl radicals for methane activation, as supported by both LIF experiments and AIMD simulations. A generalized correlation between the oxidation state of Pr in the mixed lanthanide oxide and C_2+_ yield was also observed, providing a valuable strategy for optimizing this family of OCM catalysts. Given their high yields and favorable operational parameters, Li_2_CO_3_-promoted mixed rare earth oxides hold great promise for the direct conversion of methane to C_2+_ products.

## Methods

### Redox catalyst preparation

A modified Pechini method was used to prepare LaPrO_3+x_. Stoichiometric amounts of La(NO_3_)_9_·6H_2_O (99.0%, Sigma-Aldrich, 10 g) and Pr(NO_3_)_9_·6H_2_O (99.0%, Sigma-Aldrich, 10 g) were dissolved in 100 ml deionized water and stirred to form a transparent solution. Citric acid (99.5%, Sigma-Aldrich, 28 g) at a 3:1 molar ratio to metal ions, and ethylene glycol (99.8%, Sigma-Aldrich, 18 ml) at a 2:1 molar ratio to citric acid were added into the solution. The obtained solution was stirred constantly at 80 °C to form a viscous gel. After that, the gel was transferred to a convection oven for drying at 130 °C overnight and was then calcined in a tube furnace at 850 °C for 8 h. A wet impregnation method was used to synthesize LaPrO_3+x_@Li_2_CO_3_. Calculated amount of Li_2_CO_3_ (ACS reagent; ≥99.0%) was dissolved in 10 ml deionized water. 5 g of LaPrO_3+x_ was added into the solution under stirring. After drying at 130 °C for 2 h, the dried particles were calcined in a furnace at 750 °C for 3 h. Finally, the powders were ground, pressed and crushed into 60–80 mesh as final LaPrO_3+x_@Li_2_CO_3_.

### Redox catalyst characterization

Redox catalyst surface and morphology characterizations were conducted with ex-situ and in-situ XRD, ex-situ and in-situ XPS, ex-situ and in-situ S/TEM, in-situ Raman, quasi in-situ LEIS, in-situ DRIFTS-FTIR and ex-situ XANES. Ex-situ XRD was conducted with a Rigaku SmartLab X-ray diffractometer at NC State University. In-situ XRD was conducted on an Empyrean X-ray diffractometer equipped with an Anton-Paar XRK-900 reactor chamber at NC State University. Ex-situ XPS was conducted on an ESCALAB 250Xi (Thermo Fisher) at Guangzhou Institute of Energy Conversion. In-situ XPS was conducted with SPECS EnviroESCA at Dalian Institute of Chemical Physics. S/TEM were conducted on an aberration corrected Thermo Scientific Titan 80-300 STEM at NC State University. In-situ Raman was conducted on a Horiba LabRam-HR Raman spectrometer at Lehigh University. Quasi in-situ HS-LEIS was conducted at the Surface Analysis Center at Lehigh University with an ION-TOF Qtac^100^ for outermost surface layer compositional analysis and depth profiling. In-situ DRIFTS-FTIR was conducted on a Thermo Fisher Nicolet iS50 FTIR equipped with a DiffusIR sample chamber (Pike Technologies) at NC State University. Ex-situ XANES was conducted on an X-ray Absorption Fine structure for catalysis (XAFCA) with an ion-chamber detector at Singapore Synchrotron Light Source. Characterization details of each method can be found in the supplemental document.

### Reactivity tests

Reactivity tests were conducted in a fixed bed quartz U-tube reactor with ID of 1/8 inches or 3.18 mm. Approximately 2 g of catalyst was loaded at the bottom of the U-tube reactor with quartz wool placed on both sides of the reactor to keep the catalysts in place. Typically, the OCM reaction was conducted at 700 °C, a mixture of methane (20–100%, balance Ar) was injected into the reactor for 1 min. After the OCM step, Ar was introduced to purge the reactor for 5 min and then 10% oxygen (5 mL/min, balance Ar) was introduced for the oxidation step for 3 min. A gas bag was used to collect all the gas product over the entire OCM step. The obtained gaseous products collected were detected by gas chromatography (Agilent 7890 A). To confirm the redox stability of the redox catalyst, 50 reduction and oxidation steps were performed following the above procedure, with 5 min of Ar purge in between. The catalyst OCM activity are calculated based on the average products across the OCM step obtained in the gas bag. The equations used for calculating conversions, selectivities and yields are:

1 $${{{{{\rm{Methane}}}}}}\,{{{{{\rm{Conversion}}}}}}=\frac{\,{{{{{\rm{Methane}}}}}}\,{{{{{\rm{Input}}}}}}\,-{{{{{\rm{Methane}}}}}}\,{{{{{\rm{Output}}}}}}}{{{{{{\rm{Methane}}}}}}\,{{{{{\rm{Input}}}}}}}$$

2 $${{{{{\rm{C2}}}}}}\!+\,{{{{{\rm{Selectivity}}}}}}=\frac{{{{{{\rm{moles}}}}}}\,{{{{{\rm{of}}}}}}\,{{{{{\rm{C}}}}}}\,{{{{{\rm{in}}}}}}\,{{{{{\rm{C2}}}}}}\!+\,{{{{{\rm{products}}}}}}}{{{{{{\rm{moles}}}}}}\,{{{{{\rm{of}}}}}}\,{{{{{\rm{C}}}}}}\,{{{{{\rm{in}}}}}}\,{{{{{\rm{converted}}}}}}\,{{{{{\rm{methane}}}}}}\,}$$

3 $${{{{{\rm{C2}}}}}}\!+\,{{{{{\rm{Yield}}}}}}={{{{{\rm{Methane}}}}}}\,{{{{{\rm{Conversion}}}}}}\,\ast \,{{{{{\rm{Selectivity}}}}}}\,{{{{{\rm{of}}}}}}\,{{{{{\rm{C2}}}}}}\!+$$

### Computational details

AIMD calculations were implemented by the Vienna ab initio Simulation package (VASP) with the frozen-core all-electron projector augmented wave (PAW) model and Perdew-Burke-Ernzerhof (PBE) functions. A kinetic energy cutoff of 350 eV is used for the plane-wave expansion of the electronic wave function, and a Γ-point is chosen for sampling the first Brillouin zone. The convergence criteria of force and energy were set to 0.01 eV/Å and 10^−5^ eV respectively. The strong on-site coulomb interaction on the d-orbital electrons on the Fe sites is treated with the generalized gradient approximation (GGA) + U approach with *U*_*eff*_ = 4 eV for the f-orbital of Pr. Spin polarization is included in all calculations. Constant temperature AIMD simulations are performed at 1000 K, which is slightly above the experimental reaction temperature (700 °C). The atomic motions are treated classically and propagated with 1 fs time steps.

The internal energy of all molten systems is obtained from the AIMD simulations as the time average kinetic and potential energy: $E\left(t\right)=\frac{1}{t-{t}_{0}}{\int }_{{t}_{0}}^{t}\left({E}_{{DFT}}\left(\tau \right)+{E}_{{kin}}\left(\tau \right)\right)d\tau$^6^, where ${t}_{0}$ is chosen to allow the system to equilibrate and lose memory of the initial conditions, which was set as 10 ps unless otherwise stated. For the gas-molecules, $E\left(t\right)$ are corrected by adding the translational energy $\frac{3}{2}{k}_{B}T$ because it contains only rotational and vibrational contributions, where *k*_*B*_ is the Boltzmann constant. The estimated change in Gibbs free energy is obtained as $\varDelta {G}_{{estimate}}^{^\circ }=\varDelta E+p\varDelta V-T\varDelta {S}_{{estimate}}^{^\circ }$, where the volume change ($\varDelta V$) is assumed to originate purely from changes in the number of gas phase molecules ($\varDelta {n}_{{gas}}$) and is calculated by the ideal gas law ($p\varDelta V=\varDelta {n}_{{gas}}{k}_{B}T$). The entropies of the studied radicals are obtained from NIST.

### Supplementary information

Supplementary Information

Peer Review File

### Source data

Source Data

## Data Availability

The source data generated in this study are provided in the Source Data file and are also available from the corresponding author upon reasonable request. All other data are available from the corresponding author upon request. All data needed to evaluate the conclusions in the paper are present in the paper and/or the Supplementary Materials (including Supplementary Figs. 1–15, details of the instrumentation, additional XRD, XPS, Raman and thermogravimetric analysis). The source data for the figures are all provided with this paper. Source data are provided with this paper.
