# Supplementary material for: Lithium carbonate-promoted mixed rare earth oxides as a generalized strategy for oxidative coupling of methane with exceptional yields
Source: Nat Commun. 2023 Nov 27;14:7749. doi: 10.1038/s41467-023-43682-5 (PMC10682025; doi:10.1038/s41467-023-43682-5)
Supplement: Supplementary file 1 — Supplementary Information [file 41467_2023_43682_MOESM1_ESM.pdf]

Supplementary Information (SI) for

Lithium carbonate-promoted mixed rare earth oxides as a generalized strategy for oxidative coupling of methane with exceptional yields

Kun Zhao[1,2], Yunfei Gao[3,*], Xijun Wang[4], Bar Mosevitzky Lis[5], Junchen Liu[1], Baitang Jin[1], Jacob Smith[1], Chuande Huang[6], Wenpei Gao[1], Xiaodong Wang[6], Xin Wang[3], Anqing Zheng[2], Zhen Huang[2], Jianli Hu[7], Reinhard Schömacker[8], Israel E. Wachs[4,*], Fanxing Li[1,*]

[1] North Carolina State University, Campus Box 7905, Raleigh, NC, USA

[2] CAS Key Laboratory of Renewable Energy, Guangdong Provincial Key Laboratory of New and Renewable Energy Research and Development, Guangzhou Institute of Energy Conversion, Chinese Academy of Sciences, Guangzhou, China

[3] Institute of Clean Coal Technology, East China University of Science and Technology, Shanghai, China

[4] Department of Chemical and Biological Engineering, Northwestern University, Evanston, Illinois, USA

[5] Operando Molecular Spectroscopy & Catalysis Laboratory, Department of Chemical & Biomolecular Engineering, Lehigh University, Bethlehem, PA, USA

[6] Dalian Institute of Chemical Physics, Chinese Academy of Sciences, Dalian, China

[7] Department of Chemical & Biomedical Engineering, West Virginia University, Morgantown, WV, USA

[8] Department of Chemistry, Technische Universität Berlin, Straße des 17. Juni 124, Berlin, German

Corresponding authors:

Yunfei Gao, email: yunfeigao@ecust.edu.cn

Israel E. Wachs, email: iew0@lehigh.edu

Fanxing Li, email: fli5@ncsu.ed

Contents

Details on characterization techniques 3

Ex-situ STEM-EDS on $LaPrO_{3+x}$@$5Li_2CO_3$ 5

In-situ XPS scan of Li 1s on $LaPrO_{3+x}$@$5Li_2CO_3$ 6

In-situ XRD for $LaPrO_{3+x}$ and $LaPrO_{3+x}$@$5Li_2CO_3$ 6

Additional in-situ and ex-situ Raman for $LaPrO_3$@Li_2CO_3 7

Ex-situ XANES for $LaPrO_{3+x}$ and $LaPrO_{3+x}$@Li_2CO_3 7

In-situ DRIFTS-FTIR for $LaPrO_{3+x}$@Li_2CO_3 8

Thermogravimetric analysis of $LaPrO_{3+x}$@$5Li_2CO_3$ and $LaPrO_{3+x}$..9

Structural changes of oxygen uncoupling from different materials 9

Redox OCM performance with different reduction time 9

Space-time yields with increased methane partial pressure 10

Additional ex-situ XPS for $LaPrO_3$@Li_2CO_3 10

TGA on $LaPrO_{3+x}$@$5Li_2CO_3$ using 20 redox cycles 11

Additional mass spectrometer spectrum data 12

Redox OCM performance with different Li loadings 12

Additional XRD patterns 13

Additional thermodynamic analysis 14

Details on characterization techniques:

Ex-situ XRD: Ex-situ XRD was conducted with a Rigaku SmartLab X-ray diffractometer at NC State University to analyze the crystalline phases of redox catalysts. The radiation source was a monochromatic Cu Kα (λ= 0.1542) with an operating condition at 40 kV and 44 mA. XRD patterns were generated within 2θ = 15−60° at a step size of 0.05° and a scan step time of 1 s.

In-situ XRD: In-situ XRD was conducted on an Empyrean X-ray diffractometer equipped with an Anton-Paar XRK-900 reactor chamber at NC State University. The radiation source was a monochromatic Cu Kα (λ= 0.1542) with an operating condition at 40 kV and 44 mA. XRD patterns were generated within 2θ = 15-60° at a step size of 0.05° and a scan step time of 1 s. In the temperature effect studies of $LaPrO_{3+x}$ and $LaPrO_{3+x}$@$5Li_2CO_3$, the samples were kept in an argon flow environment with a flow rate of 50 mL/min. Then, the reactor chamber was ramped up from room temperature to 700°C with a ramping rate of 5°C/min. XRD patterns were obtained during the temperature program. In the redox tests of the samples, the redox catalysts were first reduced with 10 Vol.% CH_4 (balance in Ar) for 10 mins at 700°C, and then purged with Ar for 10 mins at 700°C, and finally re-oxidized with 10 Vol.% O_2 (balance in Ar) for 10 mins at 700°C. XRD patterns were obtained during the redox cycle.

Ex-situ XPS: Ex-situ XPS was conducted with ESCALAB 250Xi (Thermo Fisher) at Guangzhou Institute of Energy Conversion. Sample powders were pressed onto a carbon tape and outgassed overnight at 10^{-5} Torr before being transferred into the analysis chamber under ultrahigh vacuum. The excitation source was a nonmonochromatic Mg Kα (1254 eV), and the XPS patterns were recorded using a PHOIBIS 150 hemispherical energy analyzer (SPECS GmbH). XPS patterns were analyzed with CasaXPS software (Casa Software Ltd., UK). A standard C 1s B.E. at 284.6 eV was used for calibration.

In-situ XPS: In-situ XPS was conducted with SPECS EnviroESCA at Dalian Institute of Chemical Physics with an Al Kα X-ray source and 20 eV pass energy. The samples were directly pressed into a 10mm-thick pellet and place into the chamber for analysis. XPS patterns were analyzed with CasaXPS software (Casa Software Ltd., UK). A standard C 1s B.E. at 284.6 eV was used for calibration. XPS peaks were first collected under ultrahigh vacuum at room temperature. Then, oxygen was dosed into the chamber until P_{O2} = 1 mbar, and XPS peaks were collected again at room temperature. The temperature of the chamber was then ramped up to 500°C under P_{O2} = 1 mbar, and held at 500°C for 30 mins before XPS peaks were collected. Then, the chamber was pumped and CH_4 was dosed into the system until P_{CH4} = 1 mbar. The temperature of the chamber was then ramped up to 700°C, and held for 10 mins before XPS peaks were collected. Then, the chamber was ramped down to 500°C and pumped again, and O_2 was dosed into the system until P_{O2} = 1 mbar. The temperature was held for 30 mins. A final XPS peak was then collected at 500° C under P_{O2} = 1 mbar.

S/TEM and EELS analysis: S/TEM were conducted on an aberration corrected Thermo Scientific Titan 80-300 STEM at NC State University. *Ex-situ STEM-EELS and ex-situ* STEM-EDS were both conducted on $LaPrO_{3+x}$@5Li_2CO_3. In-situ TEM was conducted on $LaPrO_{3+x}$@5Li_2CO_3. Basically, the sample was dispersed and then place into an environmental chip. The environmental chip was then inserted into the TEM, and 20 Vol.% O_2 (balance in Ar) was flown through the chip. The chip was then heated into 700°C under the flow of diluted O_2 and held at 700°C for analysis. In EELS analysis, the TEM sample was first prepared by dry casting the oxide particles on a carbon film supported by copper mesh grid. The sample mesh grid was then inserted into and TEM sample chamber and was vacuumed. EELS mapping was done on over the entire particle. On the edge of the particle, EELS signal mostly comes from the external surface.

Quasi in-situ HS-LEIS: Quasi in-situ HS-LEIS was conducted at the Surface Analysis Center at Lehigh University with an ION-TOF Qtac^{100} for surface compositional analysis and depth profiling. Prior to analysis, samples treated 10 Vol.% O_2 for 30 mins at 600°C in a pre-treatment chamber. After the pretreatment, the sample was cooled down under diluted O_2 and directly transferred to the analysis chamber without exposing to air. Charge neutralization was invoked during spectra acquisition and sputtering. A 3 keV He^+ (5×10^{14} cm^{-2} cyc^{-1}, 1.5 × 1.5 mm raster) was used as the detection source. A 1.0 keV Ar^+ (1.0×10^{15} cm^{-2} cyc^{-1}, 2 × 2 mm raster) was used as the sputtering source.

In-situ Raman: In-situ Raman was conducted on a Horiba LabRam-HR Raman spectrometer at Lehigh University, equipped with a 532nm excitation wavelength laser source (Oxxius, filtered to 1.5 mW) and a thermoelectrically cooled Horiba Synapse BIDD scientific CCD camera detector. The laser was focused onto the samples with a 10x objective (Olympus BX-30-LWD) while the spectrometer was used with a 500 nm grating and a 100 μm hole, resulting in a spectral resolution of ~1.7 cm^{-1}. The 520.7 cm^{-1} band of a silicon wafer standard was used to calibrate the spectrometer

prior to spectral acquisition. Powder catalysts were loaded into a quartz wool-padded reaction cell (Harrick Scientific HVC-MRA-5) and connected to a gas flow control system. A Harrick ATC Temperature Controller unit controlled the catalyst temperature and a heating/cooling rate of 10 °C min^{-1} was used throughout the *in-situ* experiments. After ambient spectra was acquired, the catalyst was dehydrated by flowing 10% O_2/Ar (30 ml/min) at 700°C for 1 hr before Raman spectra was acquired. The gas flow was then switched to 1.5% CH_4/He (30 ml/min) at 700°C for 30 mins as sample reduction step, and switched back to 10% O_2/Ar (30 ml/min) at 700°C for 30 mins as sample re-oxidation step. Raman spectra was acquired throughout.

In-situ DRIFTS-FTIR: In-situ DRIFTS-FTIR was conducted on a Thermo Fisher Nicolet iS50 FTIR equipped with a DiffusIR sample chamber (Pike Technologies) at NC State University. The samples were pressed into the sample holder and place into the *in-situ* chamber. Prior to analysis, the chamber was purged with Ar to remove CO_2 and moisture. The chamber was then heated to 650°C, held and then background was taken. 10 Vol.% O_2 (balance in Ar) was then injected for 30 mins. Then, the diluted O_2 was stopped, and the chamber was purge with Ar. After that, 10 Vol.% CH_4 (balance in Ar) was injected into the chamber. The chamber was then purged with Ar and re-oxidized with diluted O_2 again. DRIFTS-FTIR peaks were collected throughout the redox cycle.

Ex-situ XANES: Ex-situ XANES was conducted on an X-ray absorption fine structure for catalysis (XAFCA) equipment with an ion-chamber detector at Singapore Synchrotron Light Source. The electronic beam current is below 200 mA, and electronic beam energy is 700 MeV. XANES was scanned from 6420 to 6460 eV to observe the Pr L2 edges.

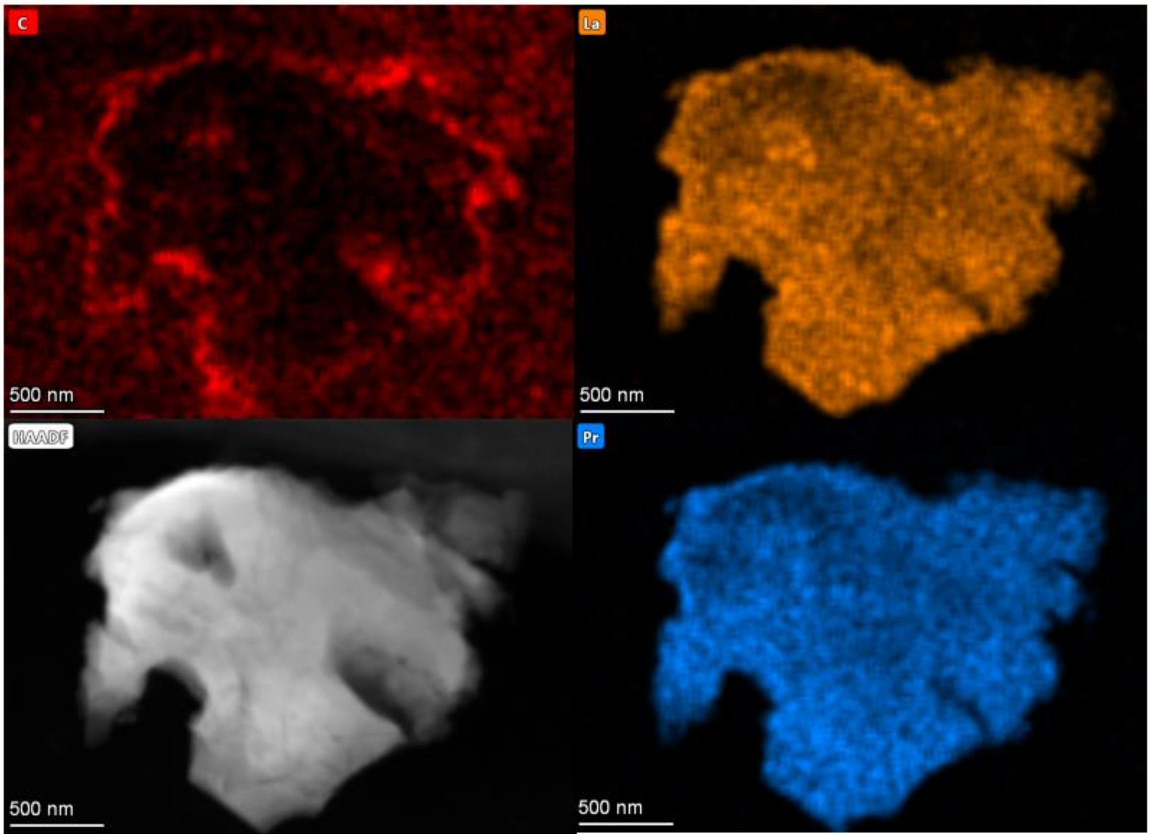

Figure S1. Ex-situ STEM-EDS on $LaPrO_{3+x}$@$5Li_2CO_3$

Fig. S1 shows the STEM-EDS of $LaPrO_{3+x}$@$5Li_2CO_3$. As can be seen La and Pr are distributed homogeneously in the material, whereas a surface enrichment of C can be observed. The EDS detector used Li drift silicon as the main part of the p-i-n junction, so detecting Li would be challenging.

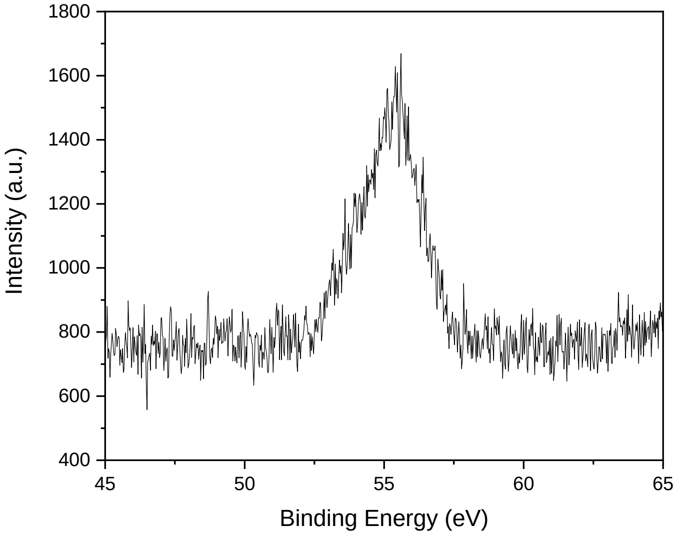

Figure S2. In-situ XPS scan of Li 1s on $LaPrO_{3+x}$@$5Li_2CO_3$ (under oxidized state)

Fig. S2 shows the in-situ XPS scan of Li 1s on $LaPrO_{3+x}$@$5Li_2CO_3$ (under oxidized state). As can be seen, the Li 1s peak can be observed, indicating the presence of surface Li.

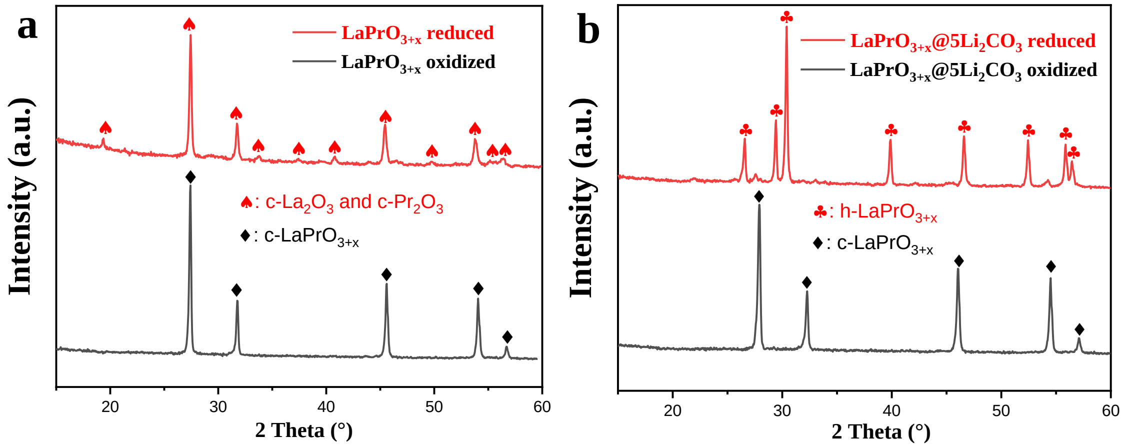

Figure S3. In-situ XRD for (a) $LaPrO_{3+x}$ and (b) $LaPrO_{3+x}$@$5Li_2CO_3$

In-situ XRD have been done on $LaPrO_{3+x}$ and $LaPrO_{3+x}$@$5Li_2CO_3$. As shown in **Fig. S3**, the oxidized Li-free bulk $LaPrO_{3+x}$ mixed oxide is present as cubic-$LaPrO_{3.33}$ and transforms to a mixture of cubic-La_2O_3 and cubic-Pr_2O_3 after methane reduction. As a comparison, the oxidized $LaPrO_{3+x}$@$5Li_2CO_3$ mixed oxide is also present as cubic-$LaPrO_{3+x}$, but transforms to the bulk hexagonal-$LaPrO_{3+x}$ phase after methane reduction.

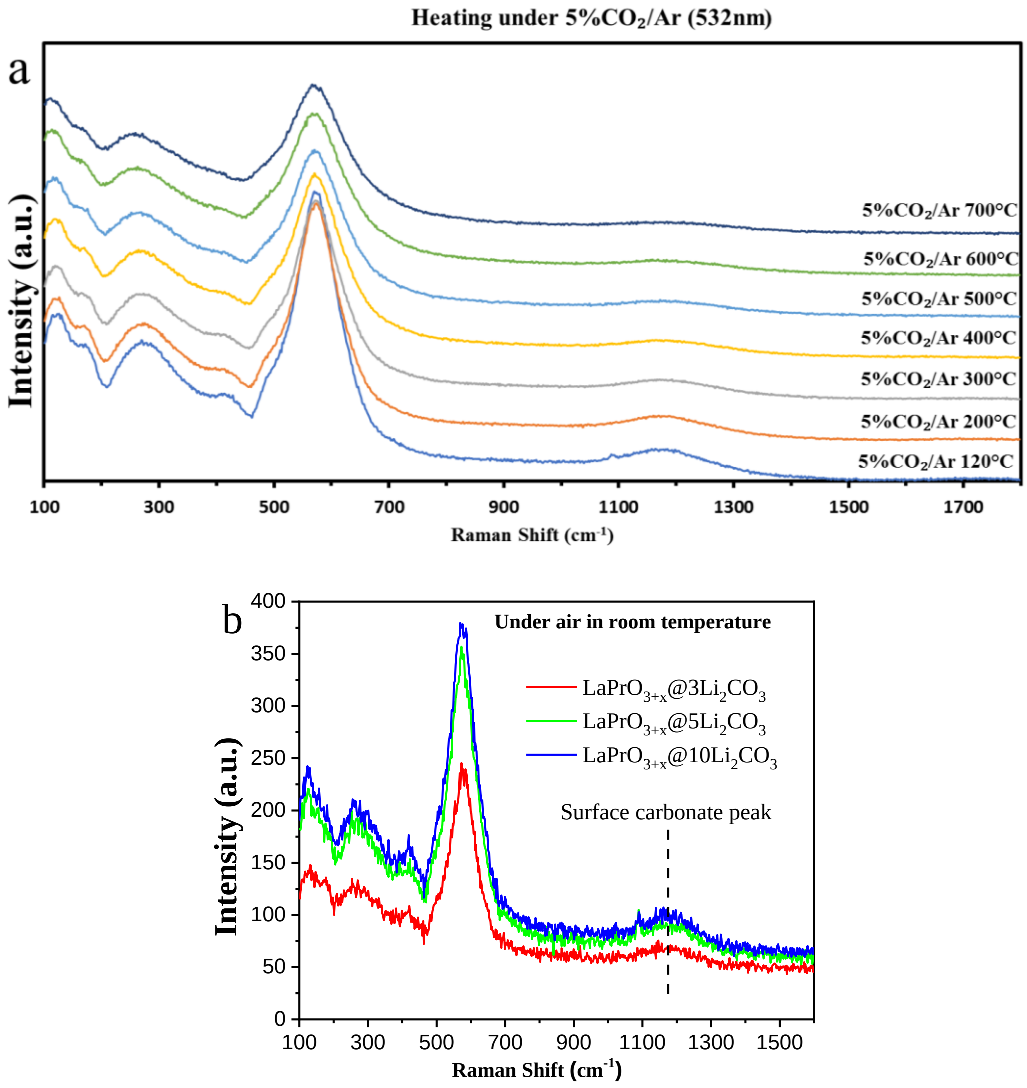

Figure S4. (a) In-situ Raman for $LaPrO_{3+x}$@$5Li_2CO_3$ under 5%CO_2/Ar; (b) Ex-situ Raman for $LaPrO_{3+x}$@$3Li_2CO_3$, $LaPrO_{3+x}$@$5Li_2CO_3$ and $LaPrO_{3+x}$@$10Li_2CO_3$ under air in room temperature.

In-situ Raman has been conducted on $LaPrO_{3+x}$@$5Li_2CO_3$ under 5%CO_2/Ar with temperature ramping up from 120 to 700°C. The results are shown in **Fig. S4(a)**. We have also compared ex-situ Raman under air in room temperature for $LaPrO_{3+x}$@$3Li_2CO_3$, $LaPrO_{3+x}$@$5Li_2CO_3$ and $LaPrO_{3+x}$@$10Li_2CO_3$. The results are shown in **Fig. S4(b)**.

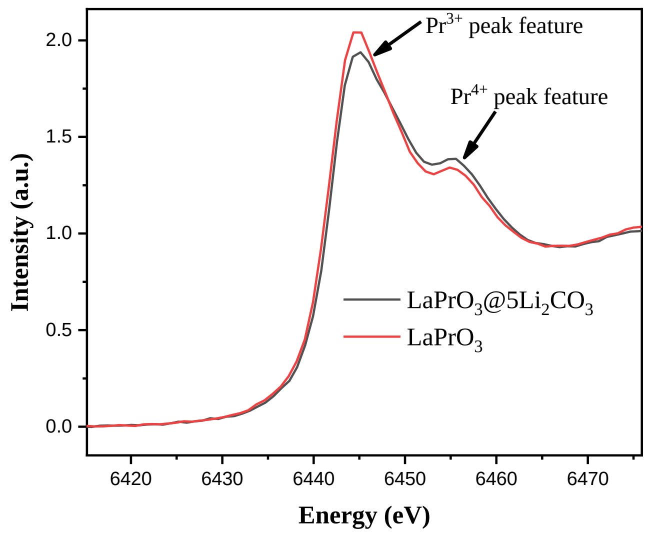

Figure S5. Ex-situ XANES on Pr L2 edge spectra of $LaPrO_{3+x}$@$5Li_2CO_3$ and $LaPrO_{3+x}$ (under oxidized state)

Fig. S5 shows the ex-situ XANES on Pr L2 edge spectra of $LaPrO_{3+x}$@$5Li_2CO_3$ and $LaPrO_{3+x}$ (under oxidized state). As reported in literature[39], the higher peak located at 6455 eV could be assigned to Pr^{4+} components and the lower peak located at 6445 eV could be assigned to Pr^{3+} components. Although the exact Pr^{3+}/Pr^{4+} component ratio is not obtained here, it is clear that $LaPrO_{3+x}$@$5Li_2CO_3$ exhibits more Pr^{4+} components, as indicated by the more intense peak at 6455 eV.

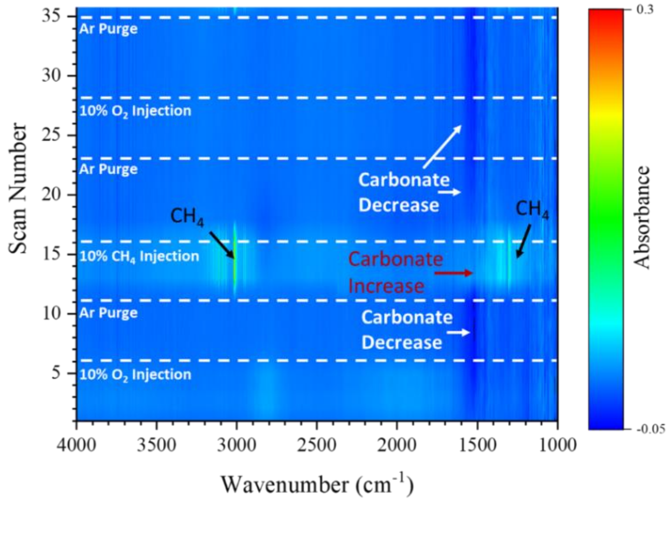

Figure S6. In-situ DRIFTS-FTIR under methane reduction, air re-oxidation and purging steps in between at 650°C

Fig. S6 shows the in-situ DRIFTS-FTIR under methane reduction, air re-oxidation and purging steps in between at 650°C. As can be seen, the carbonate signal decreases at Ar purging and oxidation steps, and increases at methane reduction steps. This indicates that methane reduction step will lead to more carbonate formation, probably due to the reaction between the partially decomposed Li_2CO_3 (which forms Li_2O) and the by-product CO_2.

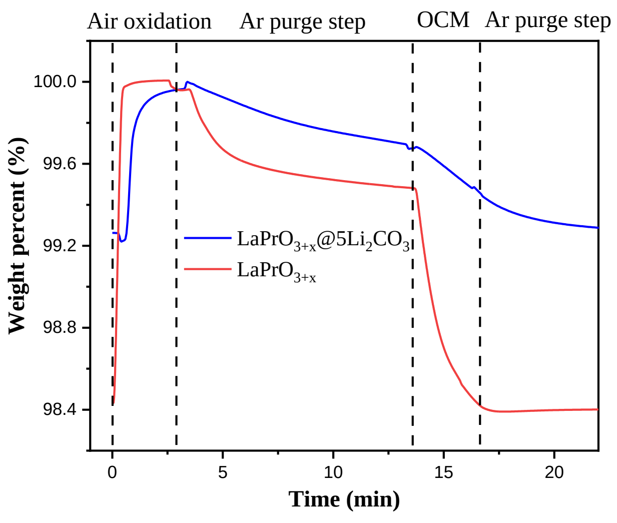

Figure S7. Thermogravimetric analysis of $LaPrO_{3+x}$@$5Li_2CO_3$ and $LaPrO_{3+x}$ under methane reduction, air re-oxidation and purging steps in between

Fig. S7 shows the thermogravimetric analysis of $LaPrO_{3+x}$@$5Li_2CO_3$ and $LaPrO_{3+x}$ under methane reduction, air re-oxidation and purging steps in between. As can be seen, the overall weight loss after methane reduction on $LaPrO_{3+x}$@$5Li_2CO_3$ and $LaPrO_{3+x}$ are 0.5 wt.% and 1.6 wt.%, respectively. Based on this value (1.6 wt.%) and assuming that Pr cations were fully reduced to +3 for $LaPrO_{3+x}$, which was suggested by XRD, the corresponding x value in $LaPrO_{3+x}$ was calculated to be 0.33. The overall weight change for $LaPrO_{3+x}$@5Li2CO3 under methane reduction (0.5 wt.%) was much smaller than that of $LaPrO_{3+x}$, indicating that the Li_2CO_3 coating inhibits the reduction of $LaPrO_{3+x}$.

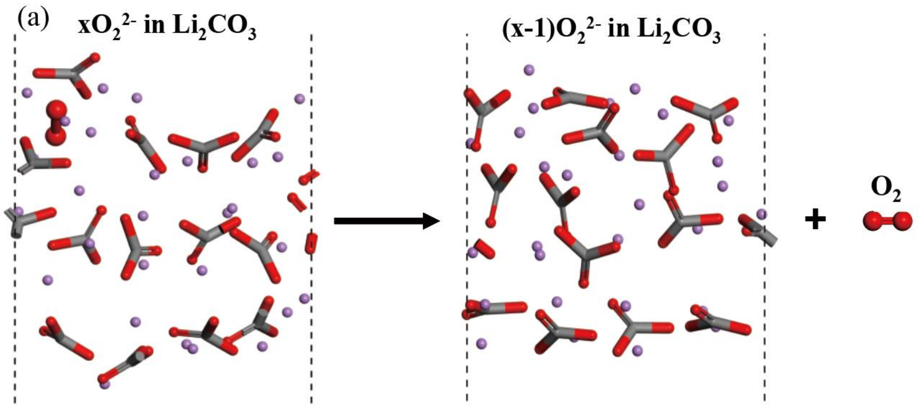

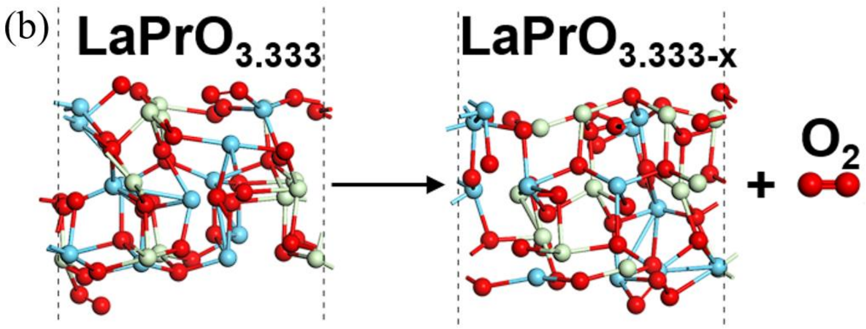

Figure. S8 The structural changes of gaseous O_2 uncoupling from (a) dissolved peroxide and (b) $LaPrO_{3.333}$

Fig. S8 shows the structural changes of gaseous O_2 uncoupling from dissolved peroxide in **Fig. S8(a)** and gaseous O_2 uncoupling from $LaPrO_{3.333}$ in **Fig. S8(b)**.

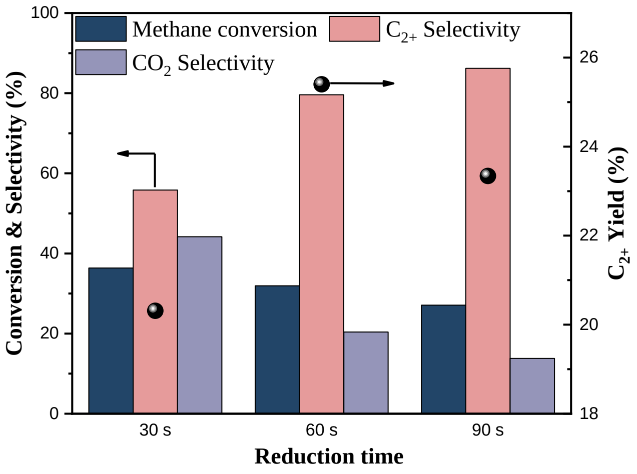

Figure S9. Redox OCM performance on $LaPrO_{3+x}$@$5Li_2CO_3$ using reduction time of 30, 60 and 90 s. Temperature = 700°C, P_{CH4} = 0.4 atm, GHSV = 1050 h^{-1}.

Fig. S9 shows the redox OCM performance on $LaPrO_{3+x}$@$5Li_2CO_3$ using reduction time of 30, 60 and 90 s, while holding the oxidation time of 180 s. As can be seen, smaller reduction time would lead to increased methane conversion but decreased C_{2+} selectivity, probably due to the more facile release of more unselective oxygen at the beginning. Larger reduction time led to decreased methane conversion but increased C_{2+} selectivity, probably due to the consumption of lattice oxygen with extended reduction time. Overall, the highest C_{2+} yield was obtained at reduction time of 60 s. We also found that the oxidation time is not as important if the period is long enough to completely regenerate the lattice oxygen. A longer oxidation time would not hurt the redox catalyst in general.

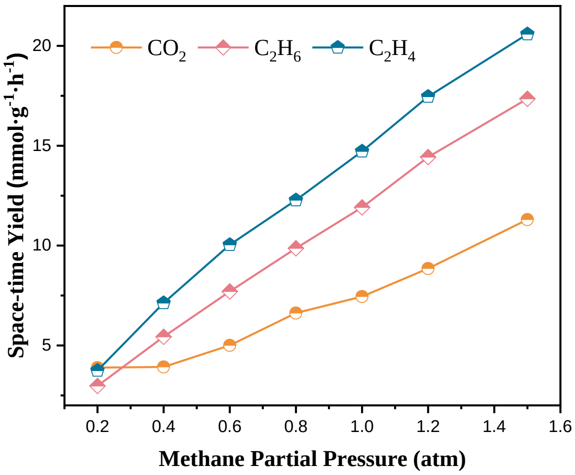

Figure S10. Space-time yields of C_2H_6, C_2H_4 and CO_2: T = 700°C, GHSV = 1050 h^{-1}

The effect of methane partial pressure was illustrated in **Fig. S10**. As can be seen, the space-time yield for ethane, ethylene and CO_2 increased almost linearly with increased methane partial pressure from 0.2 atm to 1.5 atm. This suggests a first-order kinetics for both C_2 and CO_x formation. Therefore, the $LaPrO_{3+x}$@$5Li_2CO_3$ redox catalyst can operate at elevated methane partial pressures, which would be highly beneficial for downstream separation and processing of the C_{2+} products.

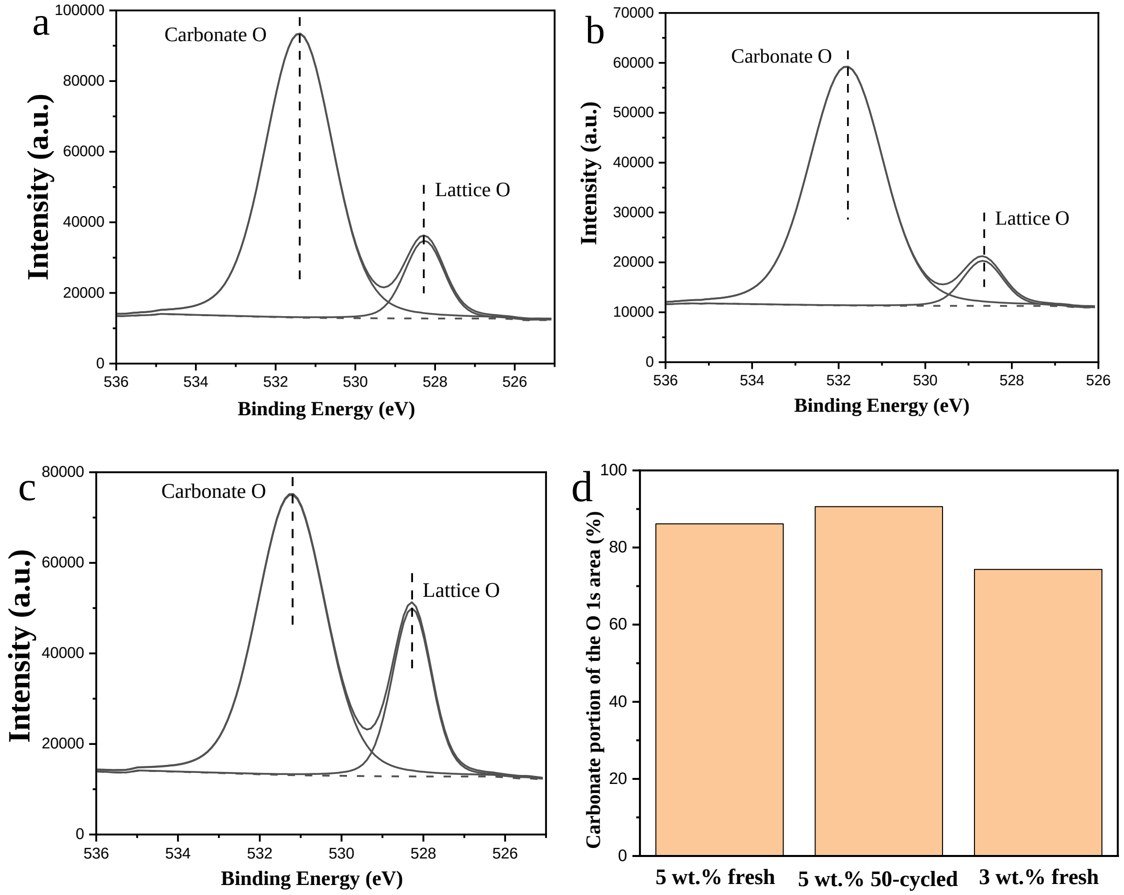

Figure S11. O 1s XPS scan on (a) 5 wt.% Li_2CO_3 coated on $LaPrO_{3+x}$, fresh sample; (b) 5 wt.% Li_2CO_3 coated on $LaPrO_{3+x}$, 50-cycled sample ended in oxidation state and (c) 3 wt.% Li_2CO_3 coated on $LaPrO_{3+x}$, fresh sample; (d) compares the carbonate O portion of the O 1s area for these samples.

Fig. S11 shows the ex-situ XPS comparison on O 1s peak of fresh $LaPrO_{3+x}$@5Li_2CO_3, $LaPrO_{3+x}$@5Li_2CO_3 after 50 redox cycles (ending in oxidation state) and fresh $LaPrO_{3+x}$@3Li_2CO_3. As can be seen, the carbonate O peak portion of the overall O peak for $LaPrO_{3+x}$@5Li_2CO_3 does not decrease after redox cycles, indicating that surface Li_2CO_3 is maintained. To confirm that the surface Li_2CO_3 content indeed has an effect on the carbonate O peak portion of the overall O peak, fresh $LaPrO_{3+x}$@3Li_2CO_3 was also compared and showed apparently decreased carbonate O.

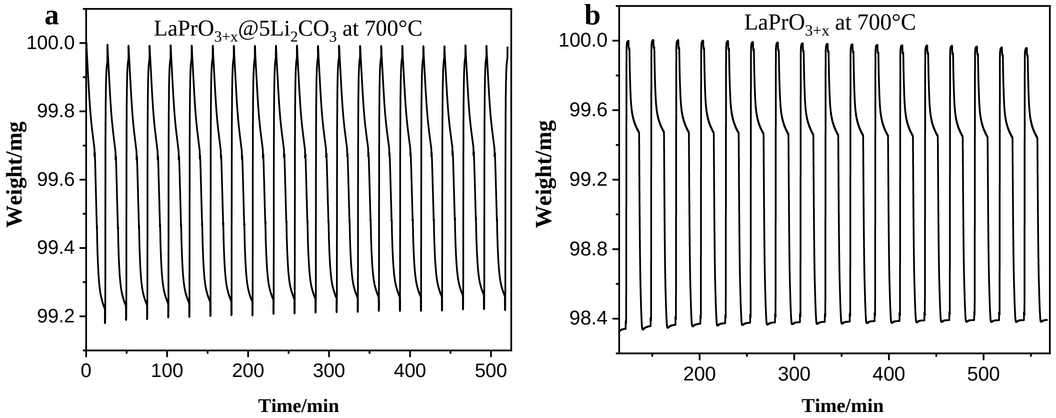

Figure S12. TGA on (a) $LaPrO_{3+x}$@5Li_2CO_3 and (b) $LaPrO_{3+x}$ using 20 redox cycles

Thermogravimetric analysis (TGA) using 20 redox cycles for $LaPrO_{3+x}$@5Li_2CO_3 were conducted as shown in **Fig. S12(a)**, and showed that the weight loss of catalysts over the redox cycles were minimal. This is vastly different from other literature reports on Li/MgO, where Li content decreased from 3.1 wt.% to ~0.1 wt.% within 20 hours.[48] The preservation of Li in $LaPrO_{3+x}$@5Li_2CO_3 is probably due to the lower reaction temperature and the dominant presence of Li_2CO_3 over LiOH with Li_2CO_3 inhibiting Li evaporation.[10] We have also conducted 20 redox cycles on bare $LaPrO_{3+x}$, as shown in **Fig. S12(b)**. As can be seen, the weight percentage change during redox cycles is ~1.6 wt.% and oxidation step also brings the mass of $LaPrO_{3+x}$ to its original value. This is consistent with O_2-TPD that Li_2CO_3 has decreased the reducibility of $LaPrO_{3+x}$.

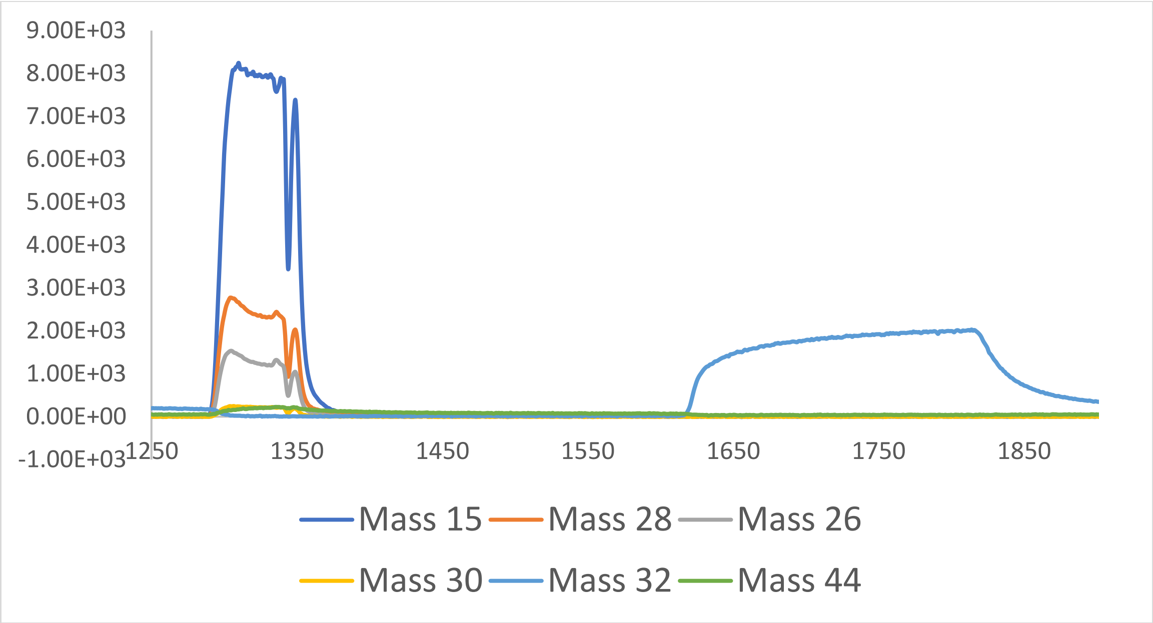

Figure S13. Mass spectrometer spectrum for an OCM redox cycle on $LaPrO_3$@$5Li_2CO_3$ after long-term cycles

Fig. S13 shows the mass spectrometer spectrum for an OCM redox cycle on $LaPrO_3$@$5Li_2CO_3$ after long-term cycles. Mass 15, Mass 26, Mass 30, Mass 32, and Mass 44 represents the characteristics peaks of CH_4, ethylene, ethane, O_2 and CO_2 respectively. We note that Mass 28 could both represent a characteristic peak of CO, or a fragment peak of ethylene or ethane. It was observed that no characteristic peaks for CO and CO_2 were observed during the re-oxidation step, indicating that the coke formation was minimal. The abrupt signal change at the end of the OCM step is due to gas switching-out.

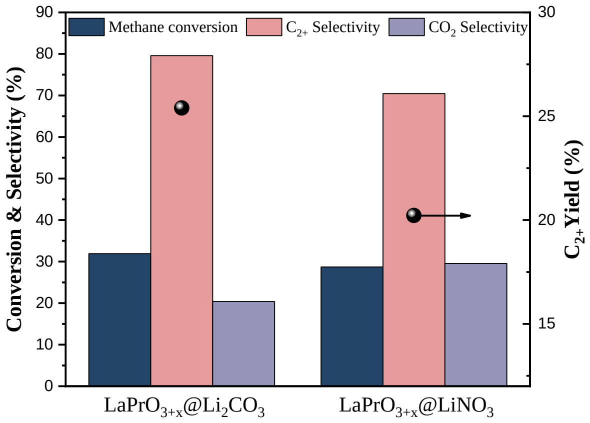

Figure S14. Redox OCM performance comparison of sintered $LaPrO_{3+x}$@Li_2CO_3 and $LaPrO_{3+x}$@$LiNO_3$ using the same Li loading. Temperature = 700°C, P_{CH4} = 0.4 atm, GHSV = 1050 h^{-1}.

Fig. S14 shows the redox OCM performance of sintered $LaPrO_{3+x}$@Li_2CO_3 and $LaPrO_{3+x}$@$LiNO_3$ using the same Li loading. As can be seen, $LaPrO_{3+x}$@$LiNO_3$ also exhibited activity for OCM, although the C_{2+} yield is lower than that of $LaPrO_{3+x}$@Li_2CO_3.

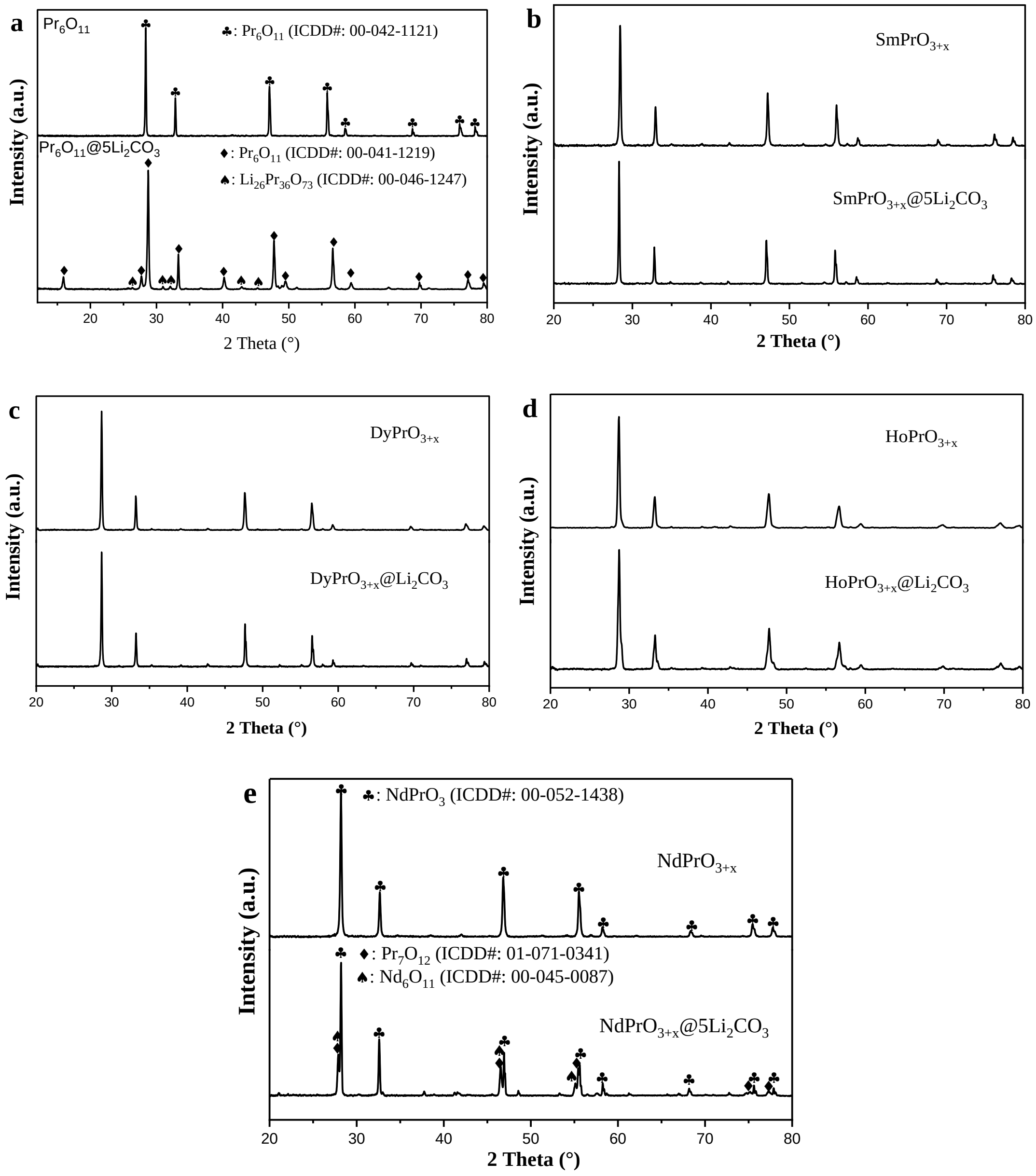

Figure S15. XRD patterns for Pr_6O_{11} and mixed metal oxides before and after Li_2CO_3 impregnation (sintered at 750°C): (a) Pr_6O_{11}; (b) $SmPrO_{3+x}$; (c) $DyPrO_{3+x}$; (d) $HoPrO_{3+x}$; (e) $NdPrO_{3+x}$

Fig. S15 shows the XRD patterns for Pr_6O_{11} and mixed metal oxides before and after Li_2CO_3 impregnation. Note that the samples were sintered again at 750°C after Li_2CO_3 impregnation. It was shown in Figure S10a that Pr_6O_{11}@5Li_2CO_3 formed a different Pr_6O_{11} phase and a secondary $Li_{26}Pr_{36}O_{73}$ phase. This would cause a decrease in Pr oxidation state and disrupt the Li_2CO_3 layer, leading to decreased C_{2+} yield. It is consistent with the study of Aono et al., where $Li_{26}Pr_{36}O_{73}$ is formed by heating Pr_6O_{11} and Li_2CO_3 at above 800°C. In fact, Aono et al. also showed that many other single rare earth metal oxides (including La_2O_3, Nd_2O_3, Sm_2O_3, Eu_2O_3, Gd_2O_3, Dy_2O_3, Ho_2O_3, Er_2O_3, Yb_2O_3, Lu_2O_3 and Y_2O_3) will form a mixed oxide phase when heating with Li_2CO_3 at above 600-900°C.[48] In comparison, all the Li_2CO_3 promoted mixed lanthanide oxides

except $NdPrO_{3+x}$ (which is not stable and partially decomposed into Pr_7O_{12} and Nd_6O_{11} at elevated temperature) have maintained their original phase after cycling (**Fig. S10b – S10e**).

Table S1. Surface areas for $LaPrO_3$, $LaPrO_{3+x}$@3Li_2CO_3, $LaPrO_{3+x}$@5Li_2CO_3 and $LaPrO_{3+x}$@10Li_2CO_3

Sample	$LaPrO_3$	$LaPrO_{3+x}$@3Li_2CO_3	$LaPrO_{3+x}$@5Li_2CO_3	$LaPrO_{3+x}$@10Li_2CO_3
Surface area (m^2/g)	0.559	1.732	1.801	1.953

Table S1 shows the surface areas for $LaPrO_3$, $LaPrO_{3+x}$@3Li_2CO_3, $LaPrO_{3+x}$@5Li_2CO_3 and $LaPrO_{3+x}$@10Li_2CO_3. As can be seen, all of these samples exhibited small surface areas in the range of 0.5-2.0 m^2/g.

Table S2. Standard Gibbs free energy change for decomposition of Li_2O_2, Na_2O_2 and K_2O_2 at 700°C

Reactions	Standard ΔG at 700°C
$2Li_2O_2 = 2Li_2O + O_2(g)$	-90.206 kJ/mol
$2Na_2O_2 = 2Na_2O + O_2(g)$	45.239 kJ/mol
$2K_2O_2 = 2K_2O + O_2(g)$	105.604 kJ/mol

Table S2 shows standard ΔG at 700°C for the decomposition of Li_2O_2, Na_2O_2 and K_2O_2 according to HSC Chemistry 9.0. As can be seen, Na_2O_2 and K_2O_2 are more stable than Li_2O_2. This might indicate lower activities for Na_2O_2 and K_2O_2 than Li_2O_2.
